# Supplementary material for: Functionally deficient UBOX5 variants and primary angle-closure glaucoma
Source: Nat Commun. 2025 Aug 15;16:7620. doi: 10.1038/s41467-025-62775-x (PMC12356834; doi:10.1038/s41467-025-62775-x)
Supplement: Supplementary file 1 — Supplementary Information [file 41467_2025_62775_MOESM1_ESM.pdf]

Supplementary Information

Supplementary Methods

Sample collections for discovery exome sequencing

Singapore: Cases of primary angle closure glaucoma (PACG) included patients of Singapore Chinese ancestry with chronic PACG or those with acute primary angle closure (APAC). All Singapore PACG cases were recruited from Singapore National Eye Centre, Tan Tock Seng Hospital, National University Hospital and Changi General Hospital. Controls were ascertained from an on-going population-based study of Chinese persons aged 40 years and older (the Singapore Chinese Eye Study [SCES]). The SCES is a population-based, cross-sectional study of Chinese adults residing in Singapore. The Ministry of Home Affairs of Singapore provided an initial computer-generated list of ethnic Chinese names of adults aged 40-80+ years of age. A final sampling frame of 6,350 ethnic Chinese residents was derived from this list using an age-stratified random sampling strategy. A control was defined as intraocular pressure (IOP) < 21 mm Hg with open angles, healthy optic nerves and normal visual fields, and no previous intraocular surgery. Ethical approval was obtained from the respective institutional review boards (IRBs).

Hong Kong: Patients with PACG were defined as above. All subjects of the Hong Kong study population were recruited from the Prince of Wales Hospital, Queen Mary Hospital, and the Hong Kong Eye Hospital, Hong Kong under the approval from the Ethics Committee on Human Research of the Chinese University of Hong Kong. A total of 575 PACG patients of Han Chinese ancestry were recruited. The controls comprised of 552 Hong Kong Chinese and were recruited in a hospital-based manner. They were all given complete ocular examinations, and confirmed to have no sign of glaucoma, angle closure or narrow angle, or other major eye diseases except for mild cataract and mild refractive errors. Control subjects were recruited from elderly people aged ≥ 60 years to ensure they were at least free of early-onset major eye diseases. They had IOP < 21 mmHg and had no known family history of glaucoma.

Vietnam: PACG cases were defined as above and were recruited from the Vietnam National Institute of Ophthalmology in Hanoi, from the Hue University of Medicine and Pharmacy in Danang City, from Viet Tiep General Hospital in Hai Phong, as well as Ho Chi Minh City Eye Hospital in Ho Chi Minh City, Vietnam. The hospital Institutional Review Boards approved the study from each site. Hospital-based controls were enrolled, and the pool of controls were further enriched by cord blood samples from the general population.

Japan: Cases of PACG in Japan were defined using the same criteria as defined above. All subjects were of Japanese descent and were recruited from Ozaki Eye Hospital in Hyuga, Oita University Hospital, Medical Foundation Tenshindo, Oita Prefectural Hospital, as well as from the University Hospital of the Kyoto Prefectural University of Medicine, Baptist Eye Clinic, and Oike Ikeda Eye Clinic (all in Kyoto city). Research protocols were approved by the IRB committee of Ozaki Eye Hospital (which includes faculty members from the Kyushu University of Health and Welfare), the Ethics Committee of Oita University Faculty of Medicine, as well as the Institutional Review Board of the Kyoto Prefectural University of Medicine. The controls were individuals aged 60 years and above without any eye diseases enrolled from the same or neighboring eye hospitals.

Sample collections for validation analysis

Brazil: PACG cases and controls were defined using the same criteria as defined above. They were recruited at the University of Campinas Clinical Hospital. Ethical approval was granted by the University of Campinas Clinical Hospital, Sao Polo, Brazil as Certificate of Presentation for Ethical Appreciaction (Certificado de Apresentação para Apreciação Ética or CAAE): 76347317.0.0000.5404 to Dr Monica Melo.

Myanmar: PACG cases and controls were defined using the same criteria as defined above. The PACG cases and controls were recruited from Defence Services General Hospital, Myanmar Eye Centre, Pun Hlaing Siloam Hospital; Shwe La Min Hospital, as well as the Mandalay Eye department, Mandalay Eye ENT hospital, University of Medicine Mandalay. The Hospital General Management committees approved the study. Final ethical approval was obtained from the Myanmar Ministry of Health.

Peru: PACG patients and controls were defined using the same criteria as defined above. They were recruited from the Instituto de Glaucoma y Catarata, Lima, Perú. Ethical approval for the study was granted by the Comité Institutional de Etica en Investigación de la Universidad de San Martín de Porres-Clínica Cada Mujer, Lima, Perú (IRB00003251-FWA0015320).

Philippines: PACG cases and controls were defined using the same criteria as defined above, and recruited at the Pasig City General Hospital, Metro Manila, the Asian Eye Institute at Makati City, and the Asian Hospital Medical

Center in Muntinlupa City The study protocol was reviewed and approved by all relevant Hospital Ethics Review Board and Ethics Review Committees.

Saudi Arabia: PACG cases and controls were defined as above. They were collected at the glaucoma clinic at the King Abdulaziz University Hospital, Department of Ophthalmology, College of Medicine, King Saud University as well as at the King Khaled Eye Specialist Hospital (both in Riyadh), Saudi Arabia. All patients and controls were unrelated Saudi Arabs, all whose known ancestors were of Saudi Arabian origin. All participants signed a written informed consent. The study was approved by College of Medicine IRB committee, King Saud University, and the IRB of the King Khaled Eye Specialist Hospital, Riyadh, Saudi Arabia.

Mexico: The PACG case and control groups were defined using the same criteria as defined above. Participants were recruited from the Department of Glaucoma, Institute of Ophthalmology "Conde de Valenciana", Mexico City, Mexico. Written informed consent was obtained from all participants. The study protocol was approved by the Hospital ethics committee.

Greece: The PACG case and control groups were defined using the same criteria as defined above. PACG cases and controls were enrolled from the University Department of Ophthalmology, Aristotle University of Thessaloniki, Thessaloniki, Greece. The control group was enriched by the inclusion of additional matched controls from the Thessaloniki Eye Study. Ethical approval was granted by the Aristotle University Medical School Ethics Committee.

Malaysia: PACG was defined using the same criteria as above. The PACG cases were recruited from the: Department of Ophthalmology, School of Medical Sciences, Universiti Sains Malaysia, Kota Bharu, Kelantan, Malaysia. This study received ethical approval from the Research and Ethical Committee, School of Medical Sciences, Universiti Sains Malaysia (USM/JEPeM/15010027) and was conducted in accordance with the Declaration of Helsinki for human research. The controls were a population-based collection of healthy ethnic Malays recruited from Singapore, as previously described.

UK Biobank collection: PACG cases in the community-based UK Biobank cohort were identified through record linkage with nationwide primary care and hospital episode databases. Participants with at least one diagnostic code for PACG (International Classification of Diseases, 9th revision: 365.2; International Classification of Diseases, 10th revision: H40.2; Read: F452) at any timepoint in their linked medical records were considered cases, with all remaining participants forming the control group. The UK Biobank was approved by the NHS North West Multicentre Research Ethics Committee (06/MRE08/65) and the National Information Governance Board for Health and Social Care. All participants provided electronic informed consent. This research has been conducted using the UK Biobank Resource under Application Number 36741.

Sample collection for de-novo validation using functional genomics

Pakistan: PACG patients and controls were defined as above. All the samples of angle closure had primary glaucoma and included patients of chronic and acute PACG. The unaffected control individuals had no such signs and symptoms, and no positive findings in their clinical histories as well as upon clinical examination. All participants were of Pakistani origin, belonging mostly to the Northern provinces of Pakistan such as Punjab and Khyber Pakhtunkhwa. All participants were recruited from Al-Shifa Eye Trust Hospital (Pakistan Institute of Ophthalmology), Rawalpindi, Pakistan, after written informed consent. The work was approved by the Ethics Review Board of the Department of Biosciences, COMSATS Institute of Information Technology, Islamabad, Pakistan in strict adherence to the tenets of the Declaration of Helsinki.

Italy: PACG patients and controls were defined as above. PACG cases were enrolled at the Dipartimento di Scienze Chirurgiche – Universita’ di Torino, Torino, Italy. Control samples were collected from the Glaucoma Center of the Ophthalmology Unit of Siena University Hospital, Siena, Italy. All patients and controls were unrelated Italians, all whose known ancestors were of Italian origin. The Comitato Etico Interaziendale A.O.U. San Giovanni Battista di Torino and the Comitato Etico of the Medical Faculty of the University of Siena, Italy, approved the study.

Whole-exome sequencing methodology

Sequencing libraries were prepared using probe-based hybridization capture kits that target the entire protein-coding genome (also known as the exome), as described elsewhere[1-3]. The libraries were then sequenced using high-throughput Illumina sequencers using 2 × 151 base-pair paired end chemistry.

Variant annotation

All identified genetic variants were annotated using GRCh37 (equivalent to hg19) database using the Ensembl Variant Effect Predictor. We annotated the following functional effects:

1. Synonymous
2. Non-synonymous
3. Splice-acceptor
4. Splice donor
5. Insertion-deletions resulting in shifting of the reading frame
6. Start loss
7. Stop loss
8. Stop gain.

Splice-site variants were only included if they affect the canonical splice sites. Finally, frame-shift variants fulfilled stringent annotation and quality metrices. For variants which have different effects across different transcripts, the highest impact effect for each variant was considered.
Non-synonymous variants are further annotated with the Combined Annotation Dependent Depletion (CADD) scaled score (phred score) software. The higher the CADD score, the more likely it is to have 'deleterious' effects. A CADD score of >10 indicates that variants predicted to be the top 10% most deleterious substitutions that can possibly occur in the human genome[4].
We adopted a minor allele frequency (MAF) threshold of <1% to define qualifying variants because variants with MAF >1% were already well represented by GWAS genotyping arrays.

Synonymous variant enrichment check to detect sequencing and analytical biases.

Synonymous variants are substitutions (usually single base pair) in the protein coding sequence that do not result in alteration of the encoded amino acid. In certain limited contexts, some synonymous variants might have functional implications due to them affecting the stability of the encoded messenger RNA. However, it is generally accepted that most synonymous variants are a neutral class of genetic variation in the context of common, complex diseases[5].

We used data on synonymous variants as a quality control measure of experimental study design. First, due to them being mostly biologically neutral, synonymous variant burden should not be significantly different between case patients and controls. This measure provides additional reassurance that potential study biases due to population stratification or analytical biases between persons with PACG and unaffected control individuals was well controlled for.

In our discovery exome-sequencing study, we did not observe any inflation of test statistics or any significant association between gene-based rare (MAF <1%) synonymous variant burden and risk of PACG (**Supplementary Figure 14**).

At *UBOX5*, we did not observe significant association between rare synonymous variant burden and risk of PACG (OR = 0.89, P = 0.8; **Supplementary Figure 15**).

Rare-variant burden analysis of genes located in the 8 loci previously reported to be associated with PACG.

There were 38 genes underlying the 8 loci previously reported to show genome-wide significant association with PACG risk. None of these 38 genes showed consistent, nominally significant differential rare variant burden between cases and controls (**Supplementary Table 8**), suggesting little overlap between common and rare variant genetic architecture for this disease.

Detailed experimental descriptions for UBOX5 biological assays

Cloning of cDNAs and plasmid construction

3X FLAG tag was attached at the 5' end of an open reading frame coding for 4 tandem repeats of the trypsin-resistant UBQLN1 ubiquitin binding Ubiquitin-associated domain (pRSET-4xTR-TUBE; a gift from Yasushi Saeki, Addgene plasmid # 110312) and its 3' end was then fused to the UBOX5 open reading frame (Genscript) by a polyglycine linker. The entire fusion construct was then cloned into the pcDNA3.1(+) vector (Genscript) by HiFi DNA assembly (NEB).

Transfection

2 ug of the construct was then transfected into 10 cm dish of HEK293 cells (~8 million cells) using the Fugene 4K reagent (E5912, Promega). 10mM Tris-HCl pH 7.5, 150mM NaCl, 0.5% NP-40, 0.5mM EDTA pH 8.0, 1mM PMSF, 10% Glycerol) with 30 μM MG132 (HY-13259, MedChem Express) and 1X Protein Phosphatase Inhibitor Cocktail (#5872, CST). Samples were sonicated at 4°C for 5 cycles (12s sonication and 20s rest) before incubation with Benzonase® Nuclease (#E1014, Sigma-Aldrich) for 2h at 4°C. Samples were then centrifuged at 21,000×g for 15 min at 4°C. The resulting supernatant was then incubated with 4μg anti-FLAG M2 (#F1804, Sigma-Aldrich) for 16 h at 4°C, and subsequently with magnetic DynabeadsTM protein G (#10004D, Invitrogen) for 1h at 4°C. After 4 washes with the lysis buffer, elution was performed by boiling the beads in elution buffer (50mM Tris-HCl pH 7.5, 1% SDS, 100mM DTT), and eluates were added to 1X Laemmli sample buffer (1610747, Bio-Rad) and sent for mass spectrometry analysis.

Mass spectrometry analysis

Immunoprecipitated samples were digested with trypsin on S-Trap□ micro columns (ProtiFi), and analysed by liquid chromatography with tandem mass spectrometry (LC-MS/MS) using an Orbitrap Eclipse Tribrid MS (Thermo Scientific) coupled to an ACQUITY UPLC M-Class (Waters) equipped with a Peptide BEH C18 column (Waters). Peptides were eluted from the column in 5-60% B (0.1% formic acid in acetonitrile) over 40 min, MS1 precursor ion spectra were acquired in the orbitrap at a resolution of 120K, and MS2 fragment ion spectra were generated using CID with fixed collision energy of 35% and acquired in the linear ion trap at 20 dependent scans per precursor. MS data was analysed using Proteome Discoverer 2.4 (Thermo Scientific) with label-free quantitation workflow and a maximum allowed fold change of 100 (by default). Proteins were identified at 1% FDR (q-value □ 0.01) with a minimum of two unique peptides per protein.

Tandem IP for validation of candidates identified by mass spectrometry analysis.

We designed a substate trapping experiment using tandem immunoprecipitation (**Supplementary Figure 6**). 6 x 10 cm dishes (~20 x 10^6 cells) were transfected with 1.8 μg of the UBOX5-UBD construct along with 2.4 μg of HA-tagged ubiquitin (pCI-neo-(HA)3-ubiquitin; a gift from Juan Bonifacino, Addgene plasmid # 196991). Cells were treated 24h after transfection with 0.7 μm Thapsigargin (#sc-24017, Santa Cruz) for a further 16h, and subsequently treated with 10 μM MG132 for another 6h before harvest. Cells were then lysed and immunoprecipitated with anti-FLAG M2 with Dynabeads™ Protein G beads as described above. Elution was then carried out by incubation with 0.2M glycine solution and neutralized with a solution of 1M Tris pH 8.5 and 1.5mM NaCl after 10mins at room temperature. 1mg/mL 3X FLAG Peptide (F4799-4MG, Sigma-Aldrich) was added to minimize rebinding of FLAG-tagged proteins to anti-FLAG M2, and 0.05% SDS added to dissociate BiP oligomers. A second immunoprecipitation was then performed using mouse anti-HA (#11583816001, Roche) and elution by heating the beads in elution buffer (62.5mM Tris-HCl pH 7.5, 1.25% SDS, 125mM DTT) and added to 1X Laemmli sample buffer as described above. The eluates were then immunoblotted using antibodies against BiP (#3177, CST), UBOX5 (NBP1-81469, Novus) and FLAG (Sigma-Aldrich).

Cellular ubiquitination assay

8 x 10^5 HEK 293 cells were transfected with 0.3 μg of Myc-tagged BiP (pCMV BiP-Myc-KDEL-wt), a gift from Ron Prywes (Addgene plasmid # 27164; http://n2t.net/addgene:27164 ; RRID:Addgene_27164), 0.2 μg HA-tagged Ubiquitin and 0.1 μg empty modified pcDNA3.1(+) vector or wildtype UBOX5 or UBOX5 variants (Twist). The pcDNA3.1(+) vector was modified by replacing the neomycin selection marker with GFP by Gibson assembly, which allowed us to compare transfection efficiency between wildtype or UBOX5 variants by immunoblotting for GFP. 24 h later, cells were treated with 0.7 μM Thapsigargin, harvested, and lysed as described above, and immunoprecipitated with antibodies against c-Myc (#sc-40, Santa Cruz). Elution was performed by heating in elution buffer (62.5mM Tris-HCl pH 7.5, 1.25% SDS, 125mM DTT) and added to 1X Laemmli sample buffer, and subsequently immunoblotted with antibodies raised against HA (#51064-2-AP, Proteintech) and c-Myc (#sc-789, Santa Cruz). 25ug of the corresponding inputs per sample were blotted for GFP (#ab13970, Abcam) and GAPDH (#sc-47724, Santa Cruz) to check for transfection efficiency. All sample inputs were blotted for GFP and GAPDH to check for transfection efficiency.

We designed an E3 ubiquitin ligase assay to test the various UBOX5 protein-altering variants, compared to wild-type UBOX5 (**Supplementary Figure 7**). MYC-Tagged BIP, wild-type UBOX5, UBOX5 variants and HA-tagged ubiquitin was co-transfected into HEK293 cells. Wild-type or variant UBOX5 were transfected. 24 hours later, cells were treated with 0.7 uM Tharpsigargin to induce ER stress. Cells were then harvested and a MYC immunoprecipitation was first performed on the lysate. Eluates were then immunoblotted with antibodies against HA to assess the amount of BIP ubiquitination.

Supplementary Table 1: Samples collections that underwent sequencing evaluations. PACG; primary angle-closure glaucoma.

Sample collection	N PACG Cases	N Controls
Discovery exome sequencing		
Singapore	2,287	1,302
Hong Kong	575	552
Japan	576	2,125
Vietnam	1,229	1,494
Total Discovery stage	4,667	5,473
Replication study		
Malays	45	709
Myanmar	103	109
Peru	77	67
Philippines	69	40
Chinese from Malaysia	113	2,136
Brazil	120	95
Saudi Arabia	162	55
Greece	36	581
Mexico	35	52
UK Biobank	1,759	467,880
Total Replication stage	2,519	471,724
De-novo validation study (informed by functional status of genetic variants)		
Italy	70	242
Pakistan	138	358
Total Confirmation study	208	600
**Total all samples studied**	**7,394**	**477,797**

Supplementary Table 2: Qualifying variant burden of *UBOX5* in the discovery exome sequencing dataset.

Coordinates	Protein alteration	N carriers in PACG cases	N carriers in Unaffected controls
**Singapore (2,287 PACG cases and 1,302 controls)**			
20:3090759:T:C	p.H540R	3	1
20:3090789:G:A	p.P530L	2	0
20:3090900:T:G	p.Y493S	9	1
20:3090934:C:T	p.E482K	1	0
20:3095974:G:C	p.S465C	1	2
20:3096055:G:A	p.T438I	1	0
20:3096091:T:C	p.K426R	1	0
20:3096109:G:A	p.P420L	1	0
20:3096110:G:A	p.P420S	1	3
20:3102335:T:C	p.Q317R	11	3
20:3102353:G:T	p.A311D	3	0
20:3102383:C:T	p.R301Q	1	0
20:3102413:T:C	p.K291R	2	0
20:3102524:T:G	p.E254A	0	1
20:3103188:C:T	p.D33N	1	0
20:3103959:T:C	p.K18R	0	1
**Total carriers in Singapore (carrier frequency)**		**38 (1.66%)**	**12 (0.92%)**
**Japan (576 PACG cases and 2,125 controls**			
20:3090789:G:A	p.P530L	0	1
20:3090934:C:T	p.E482K	2	6
20:3096005:G:A	p.Q455X	0	1
20:3096013:C:T	p.G452E	0	1
20:3096025:C:T	p.R448Q	0	1
20:3096026:G:A	p.R448W	1	0
20:3096055:G:A	p.T438I	4	6
20:3096068:C:T	p.A434T	8	5
20:3096099:G:T	p.H423Q	0	1
20:3102249:C:A	p.A346S	2	0
20:3102309:C:T	p.A326T	4	8
20:3102752:G:C	p.T178S	0	1
20:3102855:C:T	p.A144T	0	1
20:3102897:C:T	p.V130M	0	1
20:3103959:T:C	p.K18R	3	9
**Total carriers in Japan (carrier frequency)**		**24 (4.17%)**	**42 (1.98%)**
**Hong Kong (575 PACG cases and 552 controls)**			
20:3090759:T:C	p.H540R	2	2
20:3090789:G:A	p.P530L	1	0
20:3090865:C:T	p.G505S	1	0
20:3095974:G:C	p.S465C	1	0
20:3096044:T:C	p.M442V	1	0
20:3096068:C:T	p.A434T	2	0
20:3096110:G:A	p.P420S	0	1
20:3102036:A:G	p.S417P	1	0
20:3102335:T:C	p.Q317R	5	3
20:3102512:T:C	p.D258G	1	0
20:3102855:C:T	p.A144T	1	0
20:3102978:C:T	p.G103S	1	0
20:3102989:C:T	p.C99Y	1	0
20:3103031:G:A	p.T85I	0	1
20:3103059:G:C	p.Q76E	1	0
20:3103186:A:C	D33E	0	1
**Total carriers in Hong Kong (carrier frequency)**		**19 (3.3%)**	**8 (1.4%)**
**Vietnam (1,229 PACG cases and 1,494 controls**			
20:3090759:T:C	p.H540R	4	2
20:3090789:G:A	p.P530L	0	1

20:3095974:G:C	p.S465C	1	0
20:3096055:G:A	p.T438I	1	0
20:3102335:T:C	p.Q317R	19	14
20:3102735:C:T	p.G184S	3	0
20:3102777:C:T	p.V170M	1	0
20:3103188:C:T	p.D33N	1	0
20:3103959:T:C	p.K18R	2	0
**Total carriers in Vietnam (carrier frequency)**		**32 (2.6%)**	**17 (1.1%)**

Supplementary Table 3: Co-immunoprecipitation and tandem mass-spectrometry results. Listed here are the common proteins which were maximally-enriched (100× abundance ratio) and highest-scoring (top-10 Sequest HT score) across two biological replicates. All proteins were identified at false-discovery rate of 1% with a minimum of 2 unique peptides.

No.	Accession	Gene Name	Description	Replicate 1					Replicate 2				
				Score Sequest HT	# Peptides (by Search Engine)	Abundance Ratios : FLAG/IgG	Abundances (Normalized) : FLAG	Abundances (Normalized) : IgG	Score Sequest HT	# Peptides (by Search Engine)	Abundance Ratios : FLAG/IgG	Abundances (Normalized) : FLAG	Abundances (Normalized) : IgG
1	O94941	UBOX5	RING finger protein 37	353.1	23	100	1.85×10^{10}	1.08×10^{7}	133.86	21	100	3.73×10^{9}	2.30×10^{6}
2	P0DMV9	HSPA1B	Heat shock 70 kDa protein 1B	250.4	20	100	4.67×10^{9}	1.75×10^{7}	111.5	22	100	1.45×10^{9}	3.43×10^{6}
3	P11142	HSPA8	Heat shock cognate 71 kDa protein	175.76	17	100	2.14×10^{9}	1.74×10^{7}	125.32	22	100	7.95×10^{8}	3.01×10^{6}
4	P11021	HSPA5	Endoplasmic reticulum chaperone BIP	99.66	17	100	5.03×10^{8}	-	92.67	21	100	1.69×10^{8}	2.18×10^{5}
5	P0CG47	UBB	Polyubiquitin-B	95.37	5	100	2.14×10^{10}	9.65×10^{6}	62.48	5	100	4.86×10^{9}	4.65×10^{6}
	P0CG48	UBC	Polyubiquitin-C										
6	O14654	IRS4	Insulin receptor substrate 4	45.48	14	100	9.13×10^{7}	-	26.94	7	100	2.37×10^{7}	1.86×10^{5}
7	Q9Y5V3	MAGED1	Melanoma-associated antigen D1	39.31	9	100	1.12×10^{8}	-	23.02	6	100	3.11×10^{7}	-

Supplementary Table 4: List of UBOX5 protein alterations that were subjected to evaluation of the functional activity to ubiquitinate BIP. Readouts for functional assessment are obtained from Western blots appended in **Figure 4**. In the Case:Control carrier frequency ratio column, values >1 denote protein alterations that were carried more often in PACG cases compared to controls. Conversely, values <1 denote alterations that were carried more often in controls compared to PACG cases. The Combined Annotation Dependent Depletion (CADD) and Polyphen2 software are tools for scoring the deleteriousness of amino-acid substitutions (**Online Methods**). No predictions by either algorithm could be rendered for synonymous substitutions (in the case of Pro498Pro) or for deletion variants (in the case of p.Cys416del).

									Carrier frequency (%)		
**Protein Alteration**	**Sites observed**	**Functional assessment**	**CADD score**	**Polyphen2 prediction**	**N alterations in cases**	**N alterations in controls**	**N cases**	**N controls**	**Cases**	**Controls**	**Case : Control carrier frequency ratio**
p. Lys18Arg	Japan, Singapore, Vietnam	Normal Function	33	Probably Damaging	5	10	4092	4921	0.12	0.20	0.60
p. Asp33Asn	Singapore, Vietnam, UK Biobank	Normal Function	26	Probably Damaging	2	151	5275	470676	0.038	0.032	1.18
p. Val53Met	UK Biobank	Normal Function	24.3	Probably Damaging	0	69	1759	467880	0	0.00015	0
p. Thr96Met	Vietnam	Normal Function	3.8	Benign	0	1	1229	1494	0	0.067	0
p. Val109Ala	UK Biobank	Deficient	15.1	Benign	1	1	1759	467880	0.057	0.00021	266
p. Pro110Leu	UK Biobank	Deficient	19.7	Possibly Damaging	2	45	1759	467880	0.11	0.0096	11.8
p. Pro141Arg	UK Biobank	Deficient	25	Probably Damaging	1	12	1759	467880	0.057	0.0026	22.2
p. Ala144Thr	Hong Kong, Japan, UK Biobank	Normal Function	14.1	Benign	11	1365	2910	470557	0.38	0.29	1.30
p. Arg190Leu	UK Biobank	Deficient	23.4	Probably Damaging	1	15	1759	467880	0.057	0.0032	17.7
p. Ile206Lys	UK Biobank	Normal Function	24.9	Probably Damaging	0	93	1759	467880	0	0.020	0
p. Glu254Ala	Singapore	Normal function	13.3	Possibly Damaging	0	1	2287	1302	0	0.077	0
p. Glu254Lys	UK Biobank	Deficient	8.6	Probably Damaging	1	95	1759	467880	0.057	0.020	2.80
p. Lys291Arg	Singapore	Deficient	26.6	Probably Damaging	2	0	2287	1302	0.087	0	Only in cases

p. Arg301Gln	Singapore	Deficient	27.7	Probably Damaging	1	0	2287	1302	0.044	0	Only in cases
p. Ala311Asp	Singapore	Deficient	27.5	Probably Damaging	3	0	2287	1302	0.13	0	Only in cases
p. Gln317Arg	Hong Kong, Singapore, Vietnam, UK Biobank	Deficient	22.2	Benign	35	40	5850	471228	0.60	0.0085	70.5
p. Ala351Val	UK Biobank	Normal Function	19	Benign	0	83	1759	467880	0	0.018	0
p. Phe377Cys	UK Biobank	Deficient	12.5	Possibly Damaging	2	60	1759	467880	0.11	0.013	8.87
p. Cys383Ser	Vietnam	Normal Function	1.6	Benign	1	0	1229	1494	0.081	0	Only in cases
p.Cys416del	UK Biobank	Deficient	N/A (deletion)	N/A (deletion)	1	23	1759	467880	0.057	0.0049	11.6
p. Pro420Ser	Hong Kong, Singapore	Normal Function	20.1	Benign	1	4	2862	1854	0.035	0.22	0.16
p. Pro420Leu	Singapore	Normal Function	22.4	Benign	1	0	2287	1302	0.044	0	Only in cases
P. Lys426Arg	Singapore	Deficient	19.6	Benign	1	0	2287	1302	0.044	0	Only in cases
p. Thr438Ile	Japan, Singapore, Vietnam, UK Biobank	Deficient	24.2	Possibly Damaging	6	30	5851	472801	0.10	0.0063	16.2
p. Ser465Cys	Hong Kong, Singapore, Vietnam	Deficient	18.4	Benign	3	2	4091	3348	0.073	0.060	1.23
p. Leu479Pro	Common variant (gnomAD frequency >6%)	Normal Function	4.8	Benign	Common variant	Common variant	-	-	-	-	-
p. Glu482Lys	Japan, Singapore	Deficient	13.8	Benign	4	6	2863	3427	0.14	0.18	0.80
p. Arg488Ile	UK Biobank	Normal Function	23.2	Possibly Damaging	1	7	1759	467880	0.057	0.0015	38.0
p. Tyr493Ser	Singapore	Deficient	27	Probably Damaging	9	1	2287	1302	0.39	0.077	5.12
p. Pro498Leu	UK Biobank	Deficient	24.2	Possibly Damaging	1	24	1759	467880	0.057	0.0051	11.1
p. Pro498Pro	Hong Kong, Japan, Singapore, Vietnam	Normal Function	N/A (synonymous variant)	N/A (synonymous variant)	14	18	4092	4921	0.34	0.37	0.94

p. Gly514Cys	UK Biobank	Normal Function	22.4	Possibly Damaging	1	314	1759	467880	0.057	0.067	0.85
p. Pro530Leu	Hong Kong, Japan, Singapore, Vietnam	Deficient	15.8	Benign	3	2	4092	4921	0.073	0.041	1.80
p. Val536Met	UK Biobank	Deficient	22.6	Probably Damaging	1	46	1759	467880	0.057	0.0098	5.78
p. His540Arg	Hong Kong, Singapore, Vietnam	Deficient	26.2	Probably Damaging	9	5	4091	3348	0.22	0.15	1.47

Supplementary Table 5: Comparison of functional data obtained from laboratory testing and functional prediction algorithms. Variants with CADD >10 are predicted to be within the top 10% most deleterious substitutions that can occur in the human genome. For concordance comparisons, variants with CADD >10 or Polyphen2 “Damaging” predictions are expected to be of deficient function, and variants with CADD <10 or Polyphen2 “Benign” predictions are expected to be of normal function.

All variants		
	**Laboratory functional test**	
**Bioinformatic criteria**	**Normal function, N (%)**	**Deficient function, N (%)**
CADD >10	11 (73.3)	18 (90)
CADD <10	3 (20)	1 (5)
CADD no prediction	1[a] (6.7)	1[b] (5)
**Total**	**15**	**20**
Polyphen2 "Damaging"	7 (46.7)	13 (65)
Polyphen2 "Benign"	7 (46.7)	6 (30)
Polyphen2 no prediction	1[a] (6.7)	1[b] (5)
**Total**	**15**	**20**

[a] Pro498Pro synonymous variant
[b] Cys416del deletion variant

Supplementary Table 6: De-novo validation of the association between *UBOX5* coding variants and risk of PACG. The mutational landscape of *UBOX5* was surveyed in 208 patients with PACG and 600 controls from Italy and Pakistan. All coding variants detected and their distribution (variant allele counts and frequencies) in PACG cases and unaffected controls are shown. Variants qualify by bioinformatic criteria if they alter the protein coding sequence, they have allele frequency <1 percent, and have a CADD score of >10. Predictions from the Polyphen2 software, as well as variant frequency from the gnomAD database were provided for reference. Variants with deficient functional status are highlighted in red for ease of reference. The functional status of each variant can be read from **Figure 4**, relative to wild-type UBOX5. The stratified Cochran-Mantel Haenszel (CMH) test was used to meta-analyse the burden test results from Italy and Pakistan. P-values are two-sided, and not adjusted for multiple comparisons.

Variant coordinate (hg19)	Protein alteration	Variant allele count in PACG Cases, N (variant allele frequency)	Variant allele count in Unaffected Controls, N (variant allele frequency)	Qualifying variant by bioinformatic criteria	Polyphen2 prediction	CADD	gnomAD frequency	Functional status
**Italy (70 PACG cases, 242 controls)**								
20:3090826:G:A	R518C	0	1 (0.21%)	Yes	Benign	24.5	0.0016%	Normal
20:3090848:T:G	R510R (synonymous)	37 (26.8%)	152 (31.4%)	No	N/A	N/A	31.6%	Normal
20:3090855:A:C	L508R	1 (0.71%)	0	Yes	Possibly damaging	26.6	Not present	Deficient
20:3090884:C:A	P498P (synonymous)	1 (0.71%)	3 (0.62%)	No	N/A	N/A	1.2%	Normal
20:3090942:A:G	L479P	13 (9.3%)	46 (9.5%)	No	Benign	8.751	6.4%	Normal
20:3102108:G:A	P393S	0	1 (0.21%)	No	Benign	8.846	Not present	Normal
20:3102668:A:T	I206K	0	1 (0.21%)	Yes	Probably damaging	24.9	0.004%	Normal

20:3102708:C:T	V193M	0	1 (0.21%)	Yes	Probably damaging	25.2	0.0004%	Normal
20:3102842:G:A	T148I	1 (0.71%)	0	Yes	Possibly damaging	12.75	0.0008%	Deficient
20:3102855:C:T	A144T	0	2 (0.41%)	Yes	Benign	14.1	0.07%	Normal
20:3102998:G:A	T96M	1 (0.71%)	3 (0.62%)	No	Benign	3.837	0.77%	Normal
**Gene-based burden tests**								
Sum of rare missense variants		3 (2.14%)	9 (1.86%)	Odds Ratio = 1.16, *P* = 0.82				
Sum of rare missense variants with CADD >10		2 (1.43%)	5 (1.03%)	Odds Ratio = 1.39, *P* = 0.69				
Sum of rare missense variants with damaging Polyphen prediction		2 (1.43%)	2 (0.41%)	Odds Ratio = 3.53, *P* = 0.18				
Sum of functionally deficient variants		2 (1.43%)	0	Odds Ratio = 17.7, *Fexact* = 0.0498				
**Pakistan (138 PACG cases and 358 controls)**								
20:3090792:C:T	R529Q	1 (0.36%)	0	No	Benign	8.525	0.002%	Deficient
20:3090848:T:G	R510R (synonymous)	75 (27.2%)	228 (31.8%)	No	N/A	N/A	31.6%	Normal
20:3090884:C:A	P498P (synonymous)	2 (0.72%)	8 (1.1%)	No	N/A	N/A	1.2%	Normal
20:3090942:A:G	L479P	10 (3.6%)	30 (4.2%)	No	Benign	8.751	6.4%	Normal
20:3102137:C:G	C383S	4 (1.4%)	7 (0.98%)	No	Benign	3.255	0.16%	Normal
20:3102998:G:A	T96M	3 (1.1%)	3 (0.4%)	No	Benign	3.837	0.77%	Normal
20:3103188:C:T	D33N	1 (0.36%)	2 (0.28%)	Yes	Possibly damaging	26.5	0.1%	Normal
**Gene-based burden tests**								
Sum of rare missense variants		5 (1.81%)	5 (0.70%)	Odds Ratio = 2.65, *P* = 0.11				
Sum of rare missense variants with CADD >10		1 (0.36%)	2 (0.28%)	Odds Ratio = 1.3, *P* = 0.83				
Sum of rare missense variants with damaging Polyphen prediction		1 (0.36%)	2 (0.28%)	Odds Ratio = 1.3, *P* = 0.83				

Sum of functionally deficient variants	1 (0.36%)	0	Odds Ratio = 7.82, *Fexact* = 0.28
**Meta-analysis of gene-based burden tests in Italy and Pakistan (208 PACG cases and 600 controls)**			
Total sum of rare missense variants	8	14	Odds Ratio$_{\text{-CMH}}$ = 1.77 (95% ci: 0.73 – 4.30)<br>$P_{\text{-CMH}}$= 0.21
Total sum of rare missense variants with CADD >10	3	7	Odds Ratio$_{\text{-CMH}}$ = 1.36 (95% ci: 0.35 – 5.33)<br>$P_{\text{-CMH}}$= 0.66
Total sum of rare missense variants with damaging Polyphen prediction	3	4	Odds Ratio$_{\text{-CMH}}$ = 2.28 (95% ci: 0.54 – 9.72)<br>$P_{\text{-CMH}}$= 0.26
Total sum of functionally deficient alleles	3	0	Odds Ratio$_{\text{-CMH}}$ = 12.1 (95% ci: 2.2 – 66.4)<br>$P_{\text{-CMH}}$= 0.0022

Supplementary Table 7: Statistical power calculation for the discovery exome sequencing collection. The calculation was done for 4,667 persons with PACG and 5,473 unaffected individuals

CAF \ Odds ratio	1.7	1.8	1.9	2	2.1	2.2	2.3	2.4	2.5
**0.001**	0.04%	0.08%	0.14%	0.23%	0.37%	0.58%	0.88%	1.3%	1.8%
**0.0025**	0.57%	1.2%	2.3%	4%	6.7%	10.4%	15.2%	21.2%	28.2%
**0.005**	5.1%	10.5%	18.8%	29.9%	42.8%	56.2%	68.6%	78.9%	86.8%
**0.0075**	17.1%	31.4%	48.5%	65.5%	79.5%	89.1%	94.9%	97.9%	>99%
**0.01**	35.3%	56.2%	75%	88%	95.2%	98.4%	>99%	>99%	>99%
**0.0125**	55%	76.4%	90.2%	96.8%	>99%	>99%	>99%	>99%	>99%
**0.015**	71.7%	89%	96.8%	>99%	>99%	>99%	>99%	>99%	>99%
**0.0175**	83.8%	95.4%	>99%	>99%	>99%	>99%	>99%	>99%	>99%
**0.02**	91.4%	98.3%	>99%	>99%	>99%	>99%	>99%	>99%	>99%

Supplementary Table 8: Rare variant burden of genes underling the 8 previously identified GWAS loci. As per GWAS convention, the gene assigned to each loci correspond to the gene nearest to the index GWAS marker[6]. As linkage disequilibrium could be extensive, each locus could implicate more than 1 gene. All genes underlying each of the 8 previously reported loci for PACG were evaluated for rare variant burden. The statistical test used was the Sequence kernel association test (SKAT)[7] with the method set to "burden" test. SKAT is a variance component test that uses a weighted sum of chi-square statistics, which can be approximated by a chi-square distribution. The chi-square distribution is used to determine the significance of the test statistic. The P-values are two-sided and were not adjusted for multiple comparisons.

Locus	Gene	***P*-value from gene-based burden test (*P*<0.05 considered nominally significant)**			
		**Singapore**	**Hong Kong**	**Vietnam**	**Japan**
EPDR1 (Chr. 7p)	*GPR141*	0.55	0.18	0.88	0.61
	NME8	0.69	0.77	0.13	0.49
	SFRP4	0.29	0.68	1.0	0.61
	EPDR1	0.0065	0.62	0.088	0.37
	STARD3NL	0.45	1.0	0.68	1.0
GLIS3 (Chr. 9p)	*GLIS3*	0.53	0.27	0.15	0.72
CHAT (Chr. 10q)	*ERCC6*	0.31	0.0073	0.30	0.095
	PGBD3	0.92	0.24	0.74	0.56
	SLC18A3	0.18	0.63	0.26	0.18
	CHAT	0.63	0.37	0.39	0.79
	C10orf53	0.35	0.22	0.029	0.80
	ODGHL	0.30	0.11	0.86	0.77
	PARG	0.48	0.54	0.51	0.74
DPM2 – FAM102A (Chr. 9q)	*DPM2*	1.0	0.98	0.50	0.60
	FAM102A	0.57	0.63	0.16	0.14
	PIP5KL1	0.25	0.62	0.53	0.048
	ST6GALNAC4	0.51	0.94	0.28	0.79
	ST6GALNAC6	0.42	0.95	0.53	0.37
FERMT2 (Chr. 14q)	*DDHD1*	0.43	0.75	0.66	0.28
	ERO1L	0.55	0.065	0.19	0.99
	FERMT2	0.34	0.47	0.41	0.69
	GNPNAT1	0.29	0.17	0.41	0.60
	PSMC6	0.45	1.0	0.36	0.60
	STYX	0.19	0.33	1.0	0.32
PLEKHA7 (Chr. 11p)	*ABCC8*	0.50	0.53	0.68	0.46
	C11orf58	1.0	0.31	0.36	1.0
	KCNJ11	0.52	0.30	0.53	0.30
	NCR3LG1	0.27	0.69	0.43	0.41
	NUCB2	0.19	0.21	0.89	0.15
	PIK3C2A	0.0024	0.88	0.36	0.91
	PLEKHA7	0.57	0.76	0.87	0.59
	RPS13	0.45	1.0	1.0	1.0
	USH1C	0.39	0.19	0.67	0.059
COL11A1 (Chr. 1p)	*COL11A1*	0.43	0.18	0.57	0.22
PCMTD1 – ST18 (Chr. 8q)	*FAM150A*	0.060	0.59	0.84	0.12
	PCMTD1	0.19	0.98	0.045	0.46
	PXDNL	0.15	0.87	0.067	0.12
	ST18	0.94	0.80	0.48	0.28

Supplementary Figure 1: Principal component analysis of genetic ancestry for participants carrying *UBOX5* qualifying variants. The samples were enrolled from A) Singapore, B) Hong Kong, C) Japan, and D) Vietnam. Individuals carrying the *UBOX5* variants do not show evidence of bias alongside the top two principal components. Participants with PACG carrying *UBOX5* variants are colored red. Unaffected controls carrying *UBOX5* variants are colored in yellow.

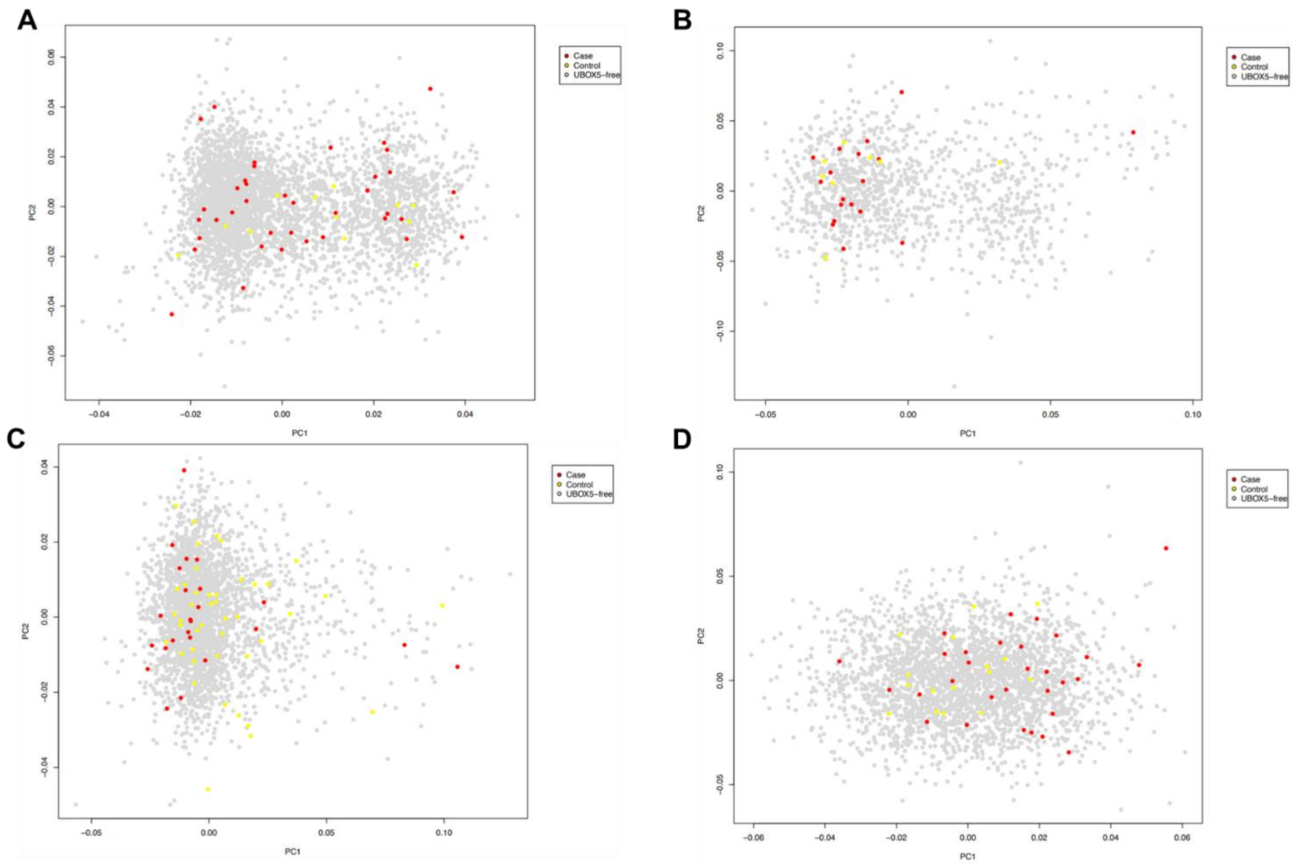

Supplementary Figure 2: Localization of UBOX5 in the iris, a major site of pathology for primary angle-closure glaucoma. UBOX5 was observed to be positively expressed (brown stains) in the sphincter pupillae of the iris.

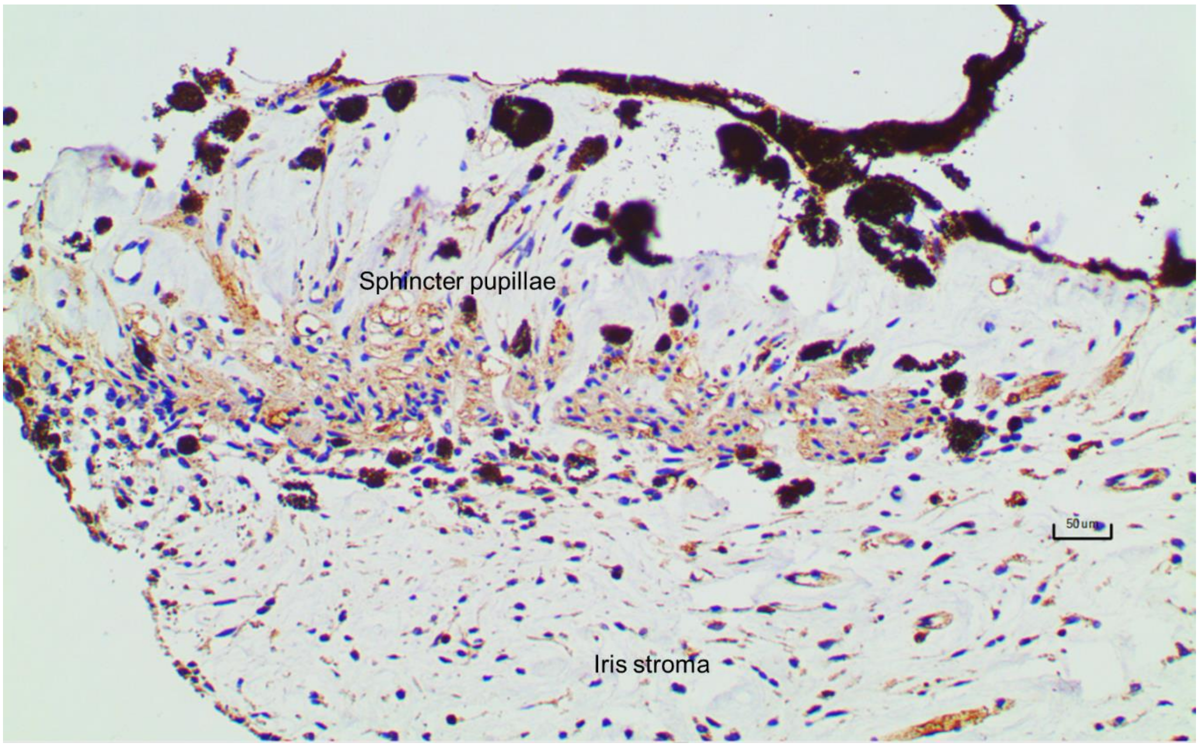

Supplementary Figure 3: UBOX5 staining in the optic nerve head (ONH) of a human sample. UBOX5 expression appears as brown stains.

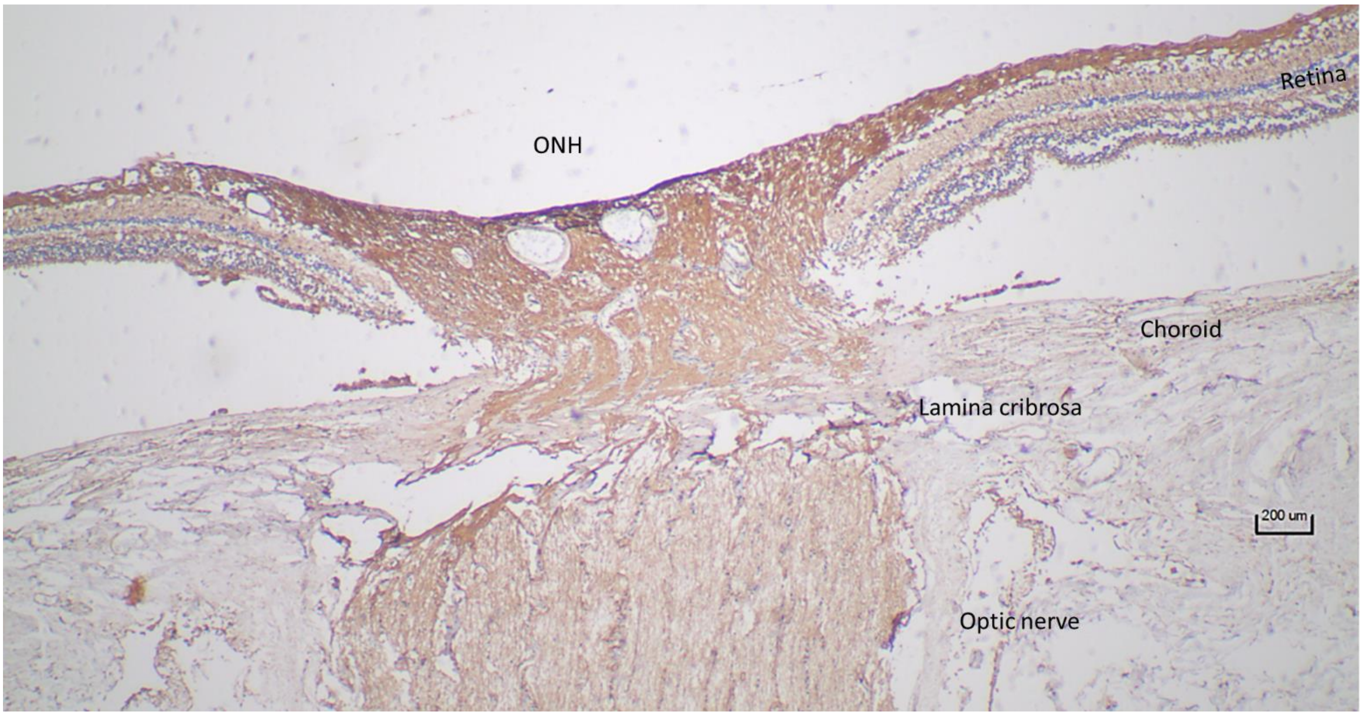

Supplementary Figure 4: UBOX5 was expressed (brown stains) in the ganglion cells (GC) of the retinal nerve fibre layer (RNFL). The yellow arrow points to amacrine cells in the inner nuclear layer (INL). The red arrow points to retinal ganglion cells (RGC).

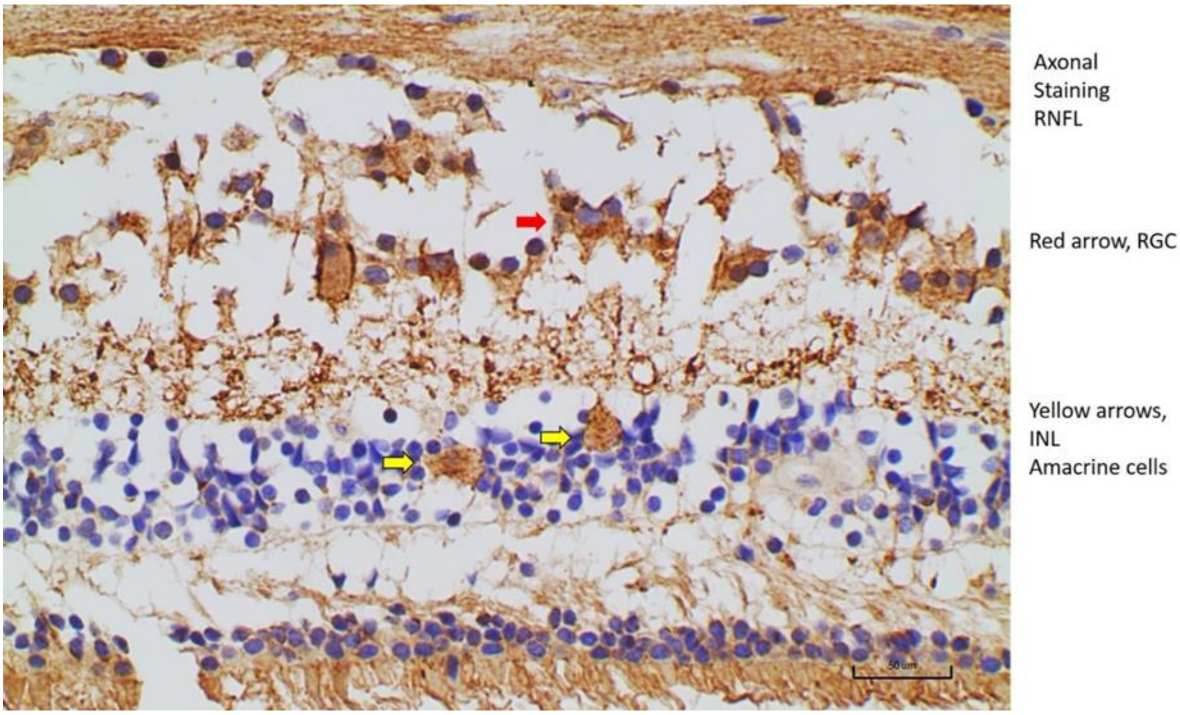

Supplementary Figure 5a: UBOX5 immunofluoresence analysis in the retina of wild-type mice (left panel) and UBOX5 knockout mice (right panel). In the wild-type mice, UBOX5 appeared to be expressed (green immunofluorescence signal) in the cytoplasm of Retinal Ganglion Cells (RGC). Within the Inner plexiform layer (IPL), the green signal highlights possible synaptic connections between the RGC, amacrine and bipolar cells in the IPL. The staining observed in wild-type mice was not observed in the retina of UBOX5 knockout mice, thus validating the specificity of the UBOX5 antibody.

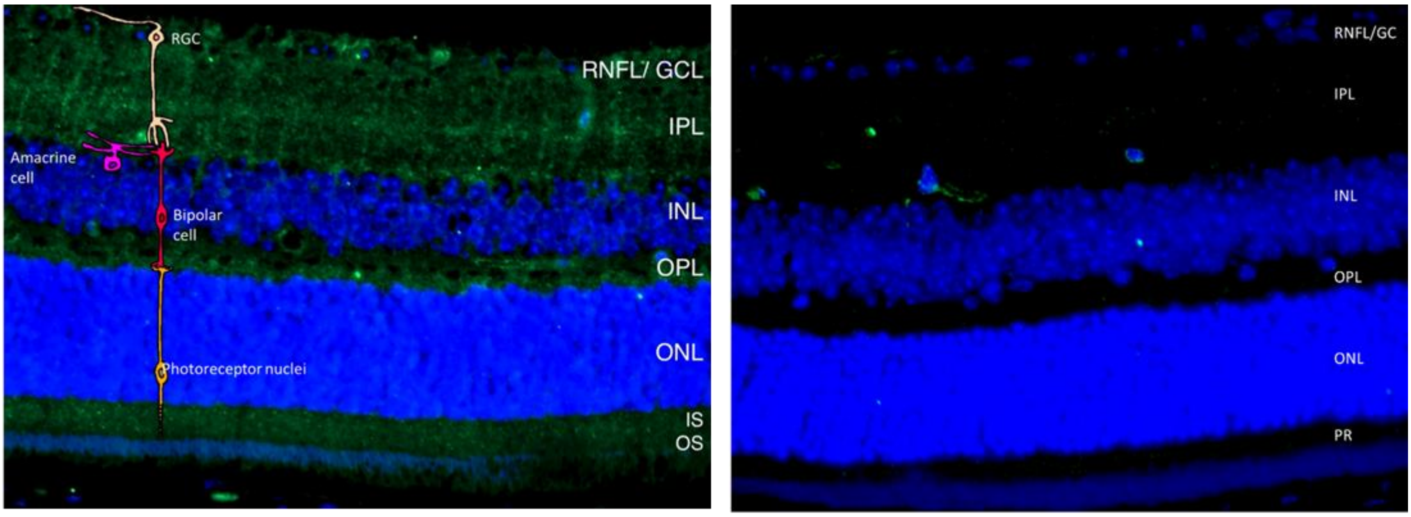

Supplementary Figure 5b: UBOX5 immunofluoresence analysis in the anterior segment of wild-type mice (left panel) and UBOX5 knockout mice (right panel). UBOX5 was positively expressed (green immunofluorescence signal) in the iris sphincter pupillae and some blood vessel muscles of the iris, as well as in the lens epithelium. Expression of UBOX5 was abolished in the anterior chamber tissues of UBOX5 knockout mice thus validating the specificity of the UBOX5 antibody.

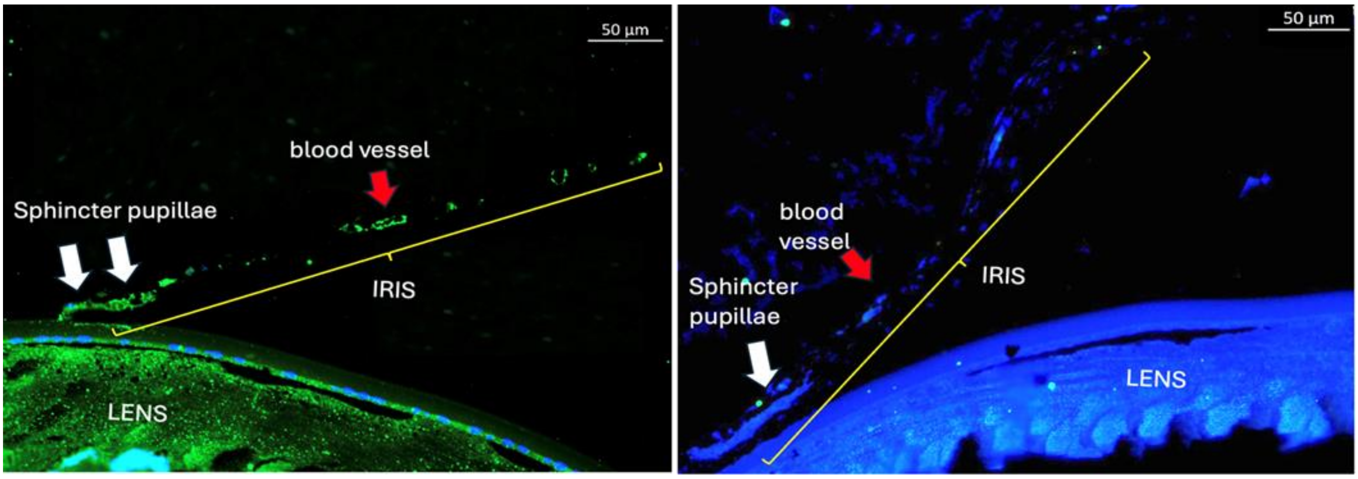

Supplementary Figure 6: Diagram showing the design of the substrate trapping for the tandem immunoprecipitation experiment. Created in BioRender. Wang, Z. (2025) https://BioRender.com/j67z6n6

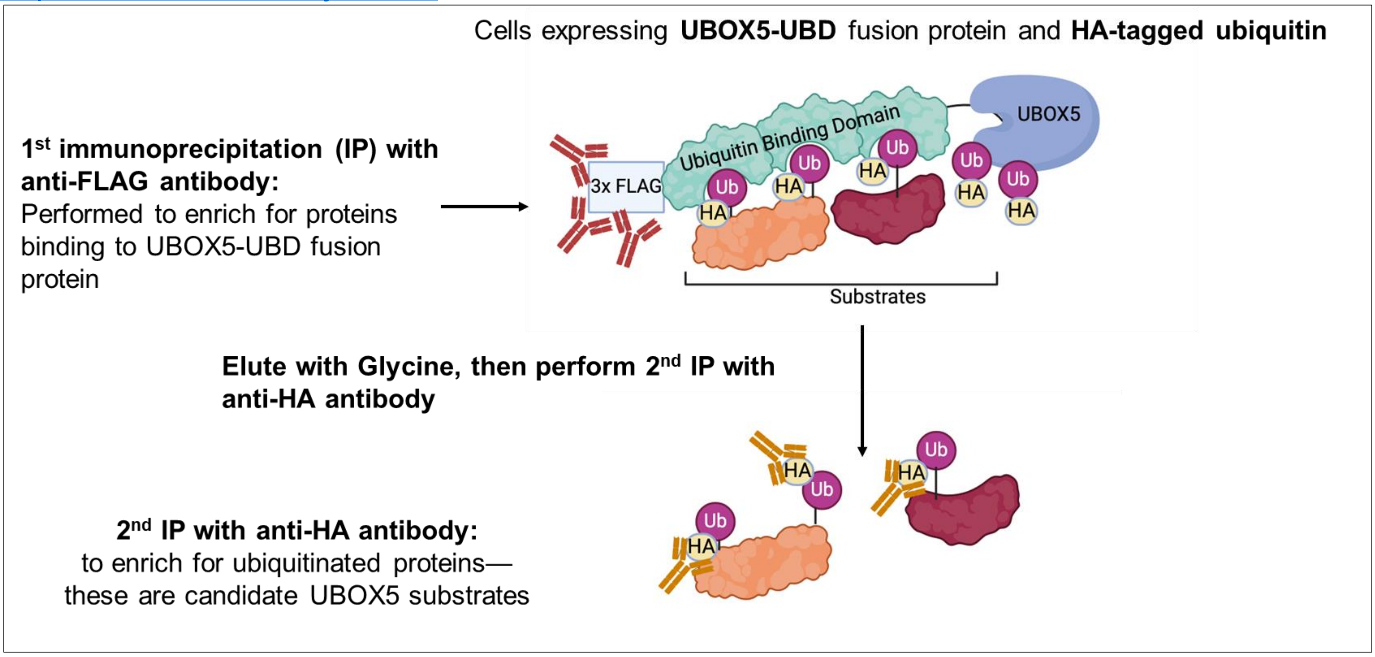

Supplementary Figure 7: Diagram showing the design of the E3 ubiquitin ligase assay for UBOX5 variants. Created in BioRender. Wang, Z. (2025) https://BioRender.com/7h2j30o

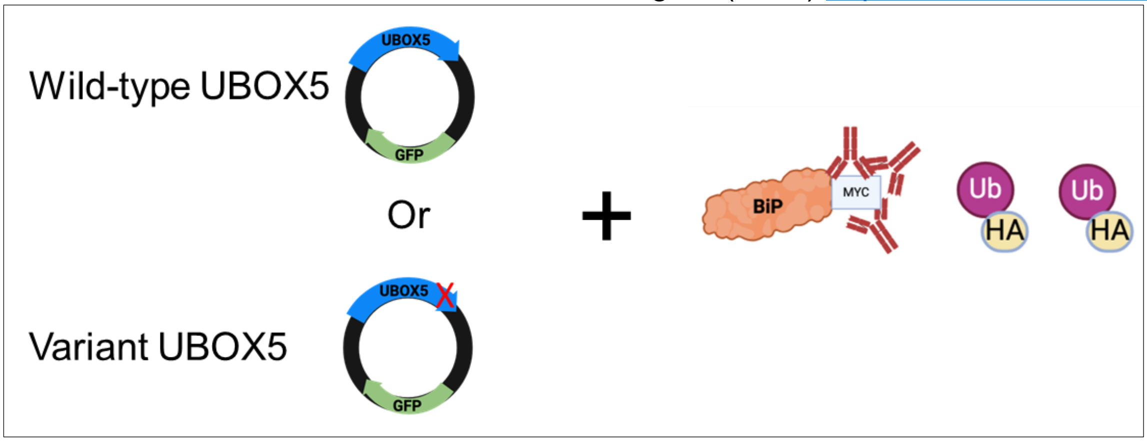

Supplementary Figure 8: Functional activity of the different UBOX5 variants shown in separate Western blot gels. Full gels are shown, with no splicing of lanes. MYC-Tagged BIP, wild-type UBOX5, UBOX5 variants and HA-tagged ubiquitin was co-transfected into HEK293 cells. Wild-type and variant UBOX5 were transfected in their native form. 24 hours later, cells were treated with 0.7 uM Tharpsigargin to induce ER stress. Cells were then harvested and a MYC immunoprecipitation was performed on the lysate. Eluates were immunoblotted with antibodies against HA to assess the amount of BIP ubiquitination. Immunoprecipitation efficiency was assessed by MYC immunoblot after stripping the membrane. Transfection efficiency was assessed by GFP abundance in input lysates. Both measures of efficiency must be similar between wild-type (reference) and variant (test) UBOX5 alleles before functional comparisons can be made. **Wild-type UBOX5 alleles are taken as reference and are shown in blue**. Alleles unique to the de-novo validation study in Italy and Pakistan are shown in **red**. For added experimental vigour, alleles from the de-novo validation study are run across different batches so that multiple comparisons against multiple independent wild-type UBOX5 alleles can be made. **The intensity of the IB:HA band correlates directly with the amount of ubiquitin deposited by UBOX5 onto BIP**. Functionally deficient UBOX5 variants will deposit less ubiquitin onto BIP, thus resulting in a fainter band compared to wild-type UBOX5.

NF denotes normally functioning alleles whereby the intensity of the test allele's band appeared similar to that of wild-type UBOX5. D denotes functionally deficient alleles whereby the intensity of the test allele's band appeared clearly weaker by eye to that of wild-type UBOX5.

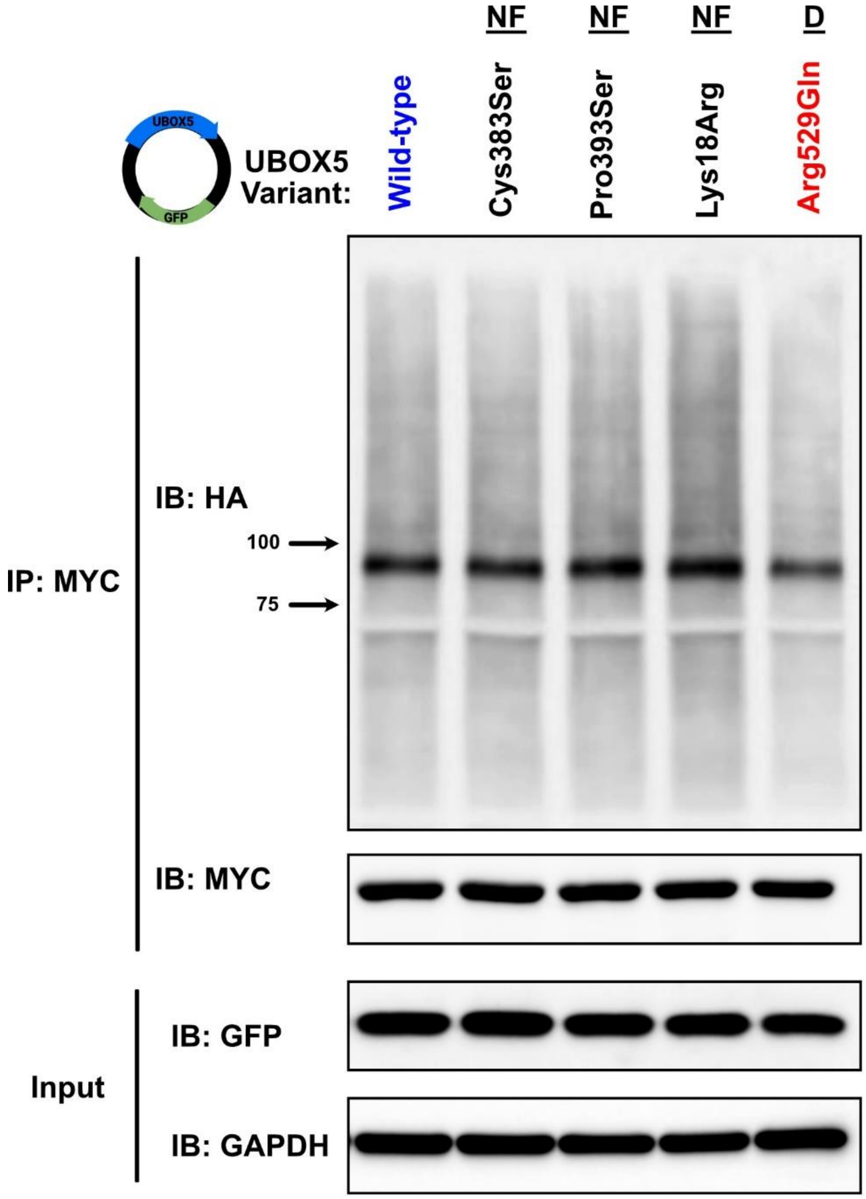

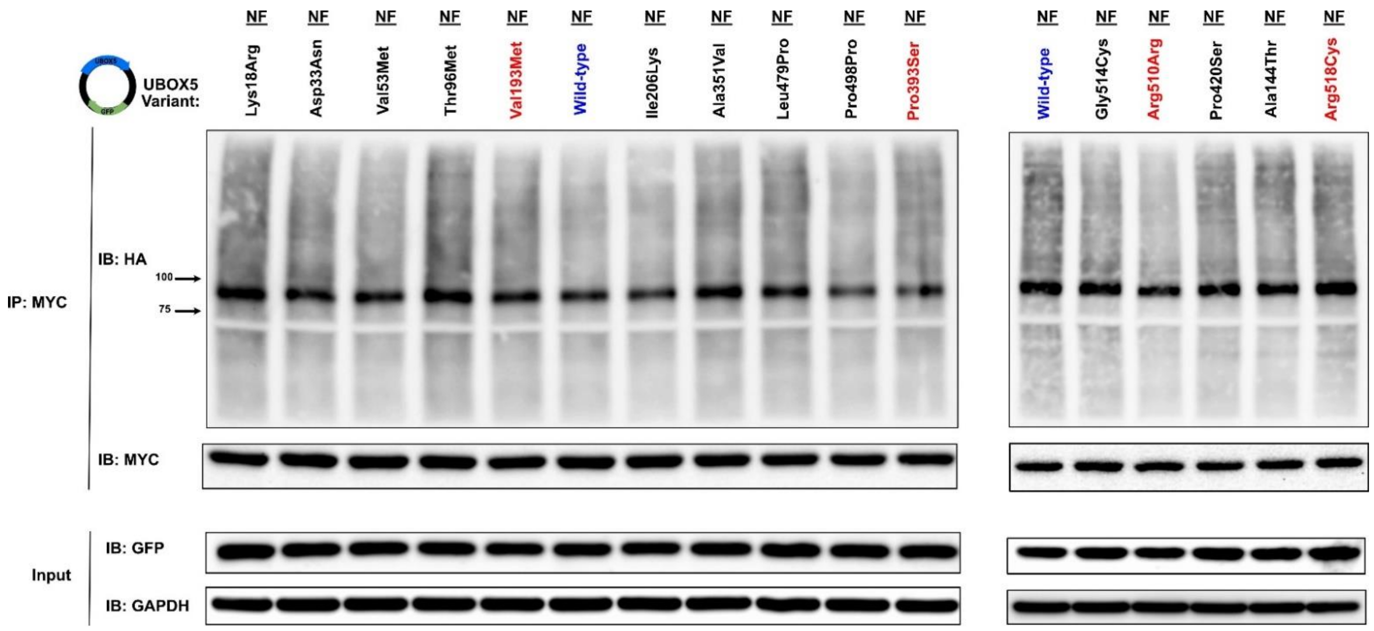

NF NF NF NF NF NF NF NF NF NF NF
UBOX5 Variant:
Lys18Arg
Asp33Asn
Val53Met
Thr96Met
Val193Met
Wild-type
Ile206Lys
Ala351Val
Leu479Pro
Pro498Pro
Pro393Ser
NF NF NF NF NF NF
Wild-type
Gly514Cys
Arg510Arg
Pro420Ser
Ala144Thr
Arg518Cys
IP: MYC
IB: HA
100
75
IB: MYC
Input
IB: GFP
IB: GAPDH

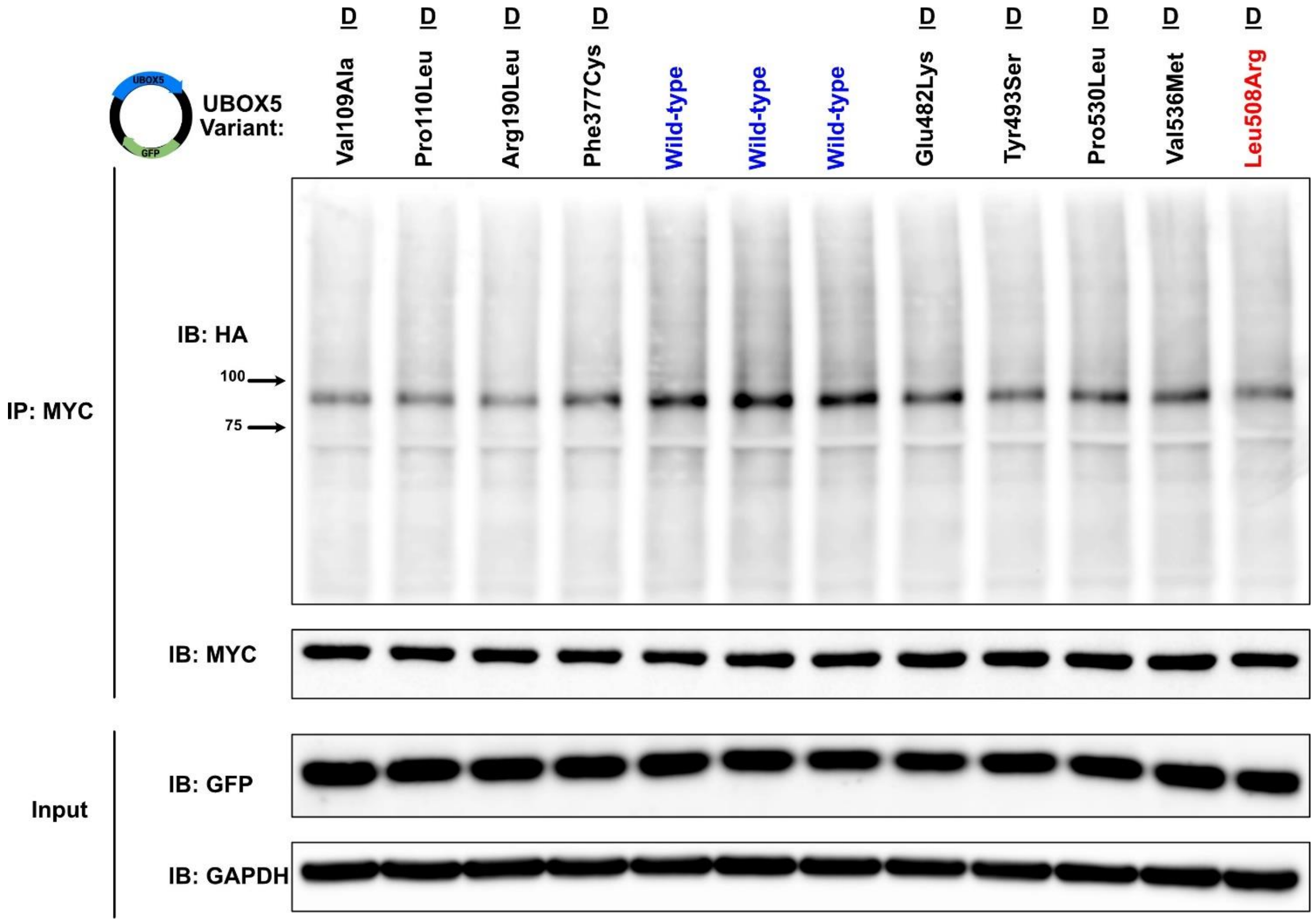

D D D D D D D D D
UBOX5 Variant:
Val109Ala
Pro110Leu
Arg190Leu
Phe377Cys
Wild-type
Wild-type
Wild-type
Glu482Lys
Tyr493Ser
Pro530Leu
Val536Met
Leu508Arg
IP: MYC
IB: HA
100
75
IB: MYC
Input
IB: GFP
IB: GAPDH

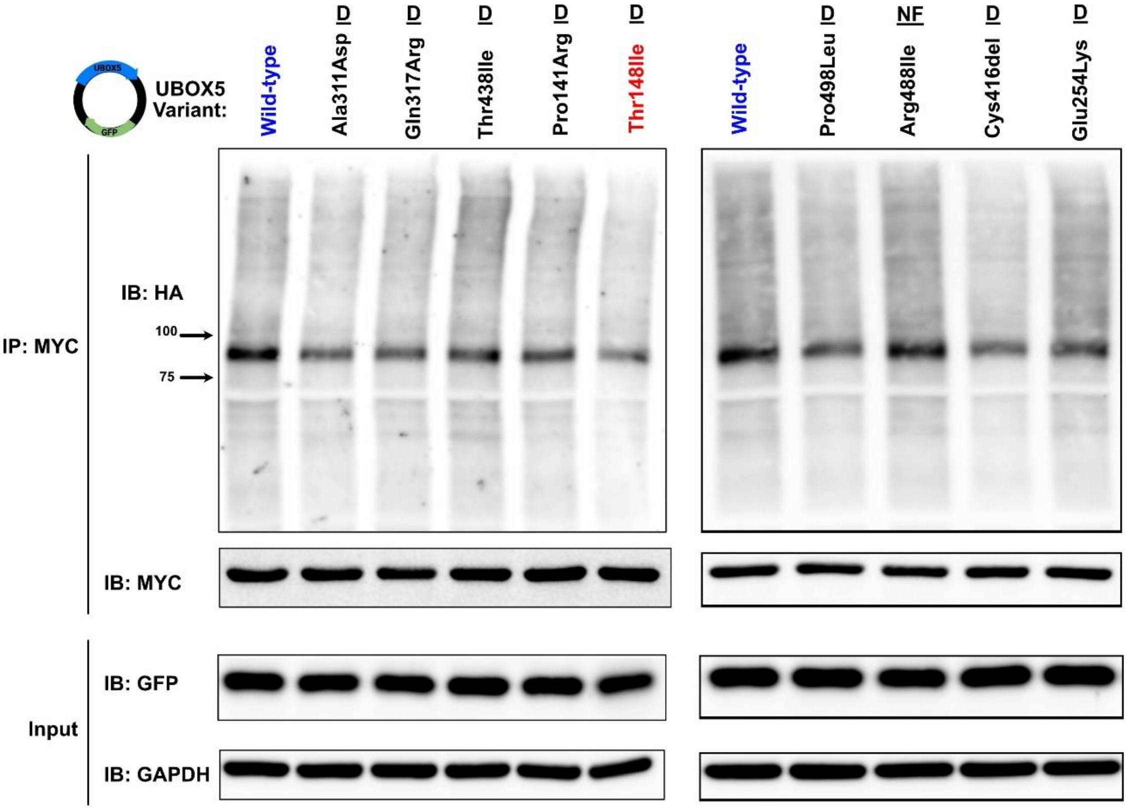
UBOX5
GFP
UBOX5 Variant:
Wild-type
D
Ala311Asp
D
Gln317Arg
D
Thr438Ile
D
Pro141Arg
D
Thr148Ile
Wild-type
D
Pro498Leu
NF
Arg488Ile
D
Cys416del
D
Glu254Lys
IP: MYC
IB: HA
100
75
IB: MYC
Input
IB: GFP
IB: GAPDH

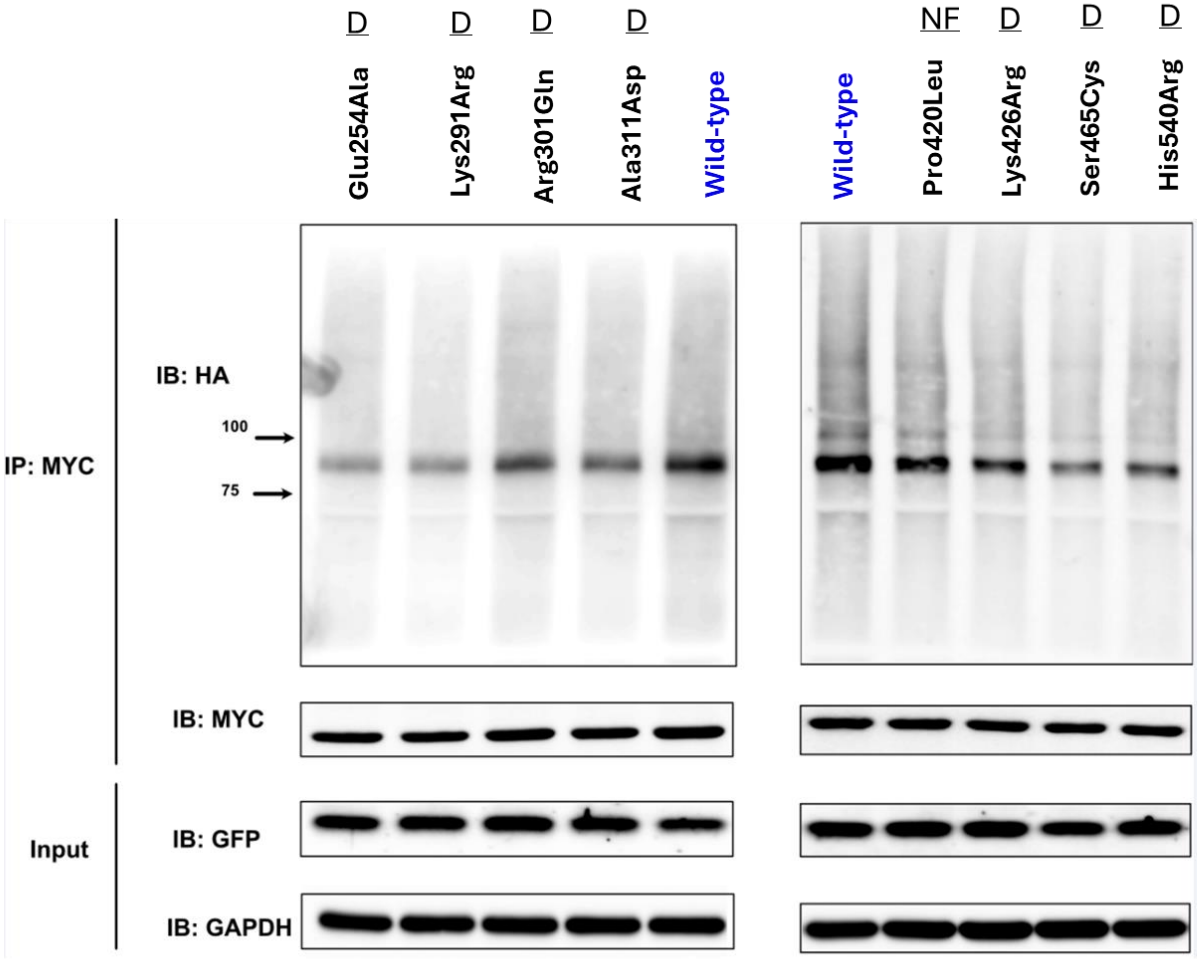
D
Glu254Ala
D
Lys291Arg
D
Arg301Gln
D
Ala311Asp
Wild-type
Wild-type
NF
Pro420Leu
D
Lys426Arg
D
Ser465Cys
D
His540Arg
IP: MYC
IB: HA
100
75
IB: MYC
Input
IB: GFP
IB: GAPDH

Supplementary Figure 9: Number of singleton variants for each sample in the discovery exome sequencing analysis, stratified by country of enrollment.

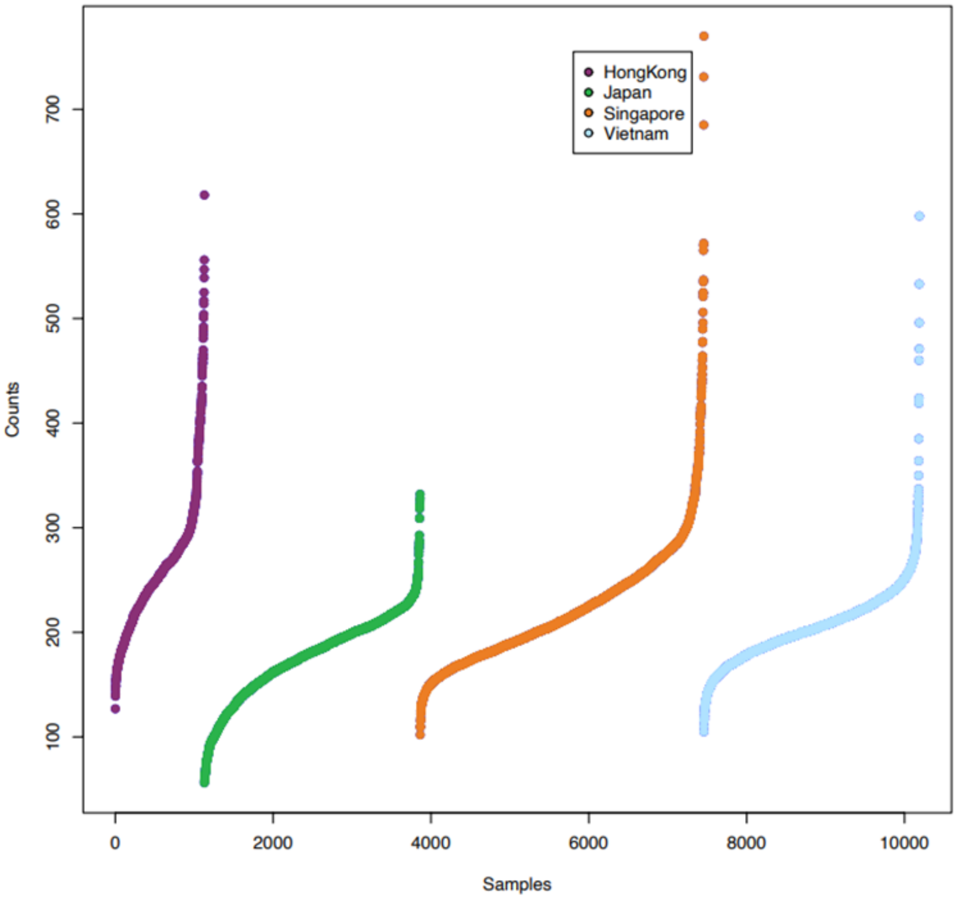

Supplementary Figure 10: Principal component analysis of genetic ancestry for the discovery exome sequencing study. The samples were enrolled from A) Singapore, B) Hong Kong, C) Japan, and D) Vietnam.

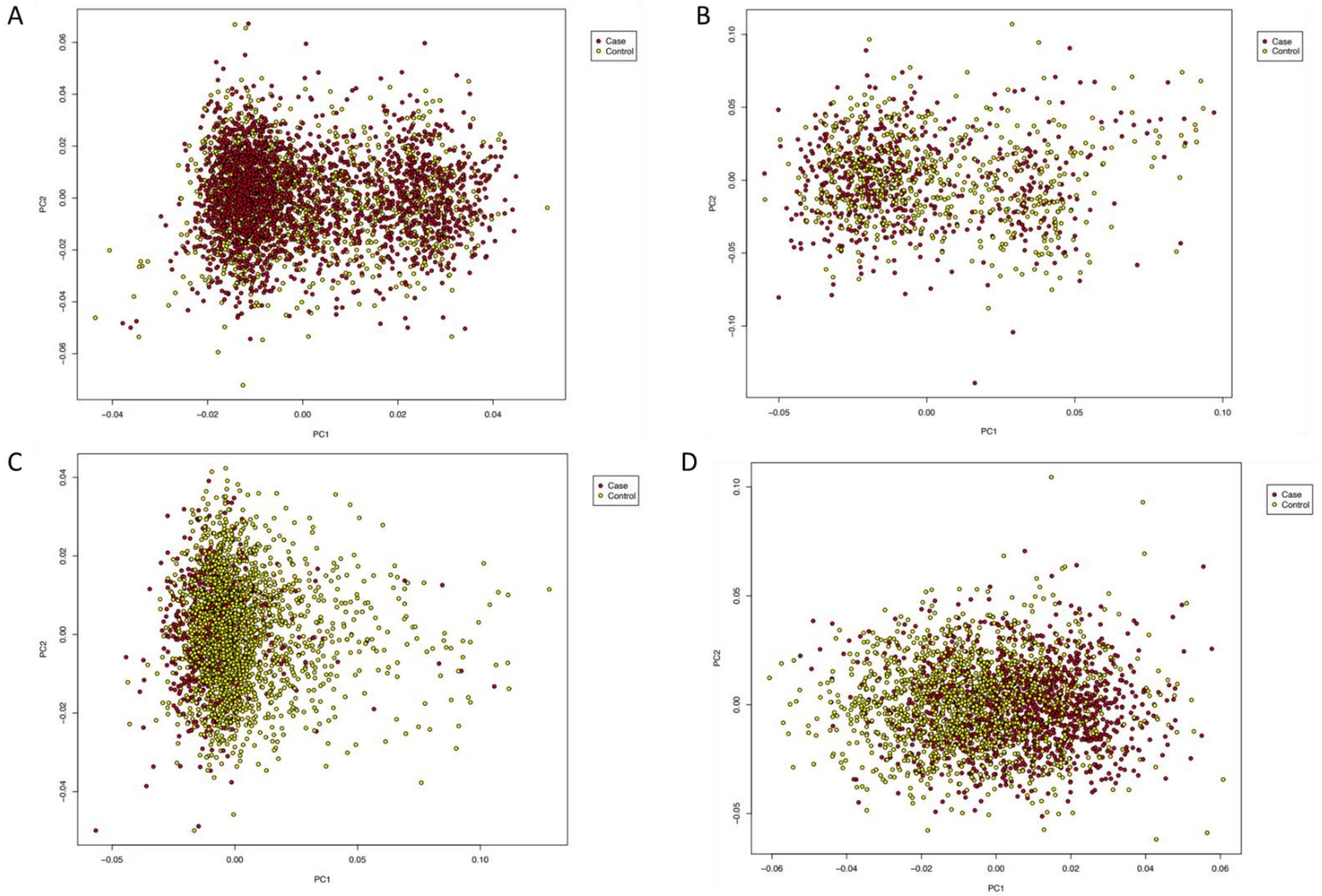

Supplementary Figure 11: Capillary sequencing traces (using the Sanger method) for a selection of *UBOX5* qualifying variants.

UBOX5 p.K291R

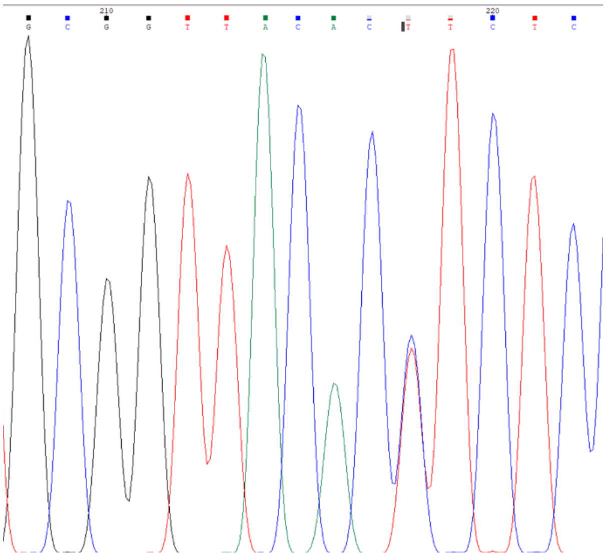

UBOX5 p.H540R

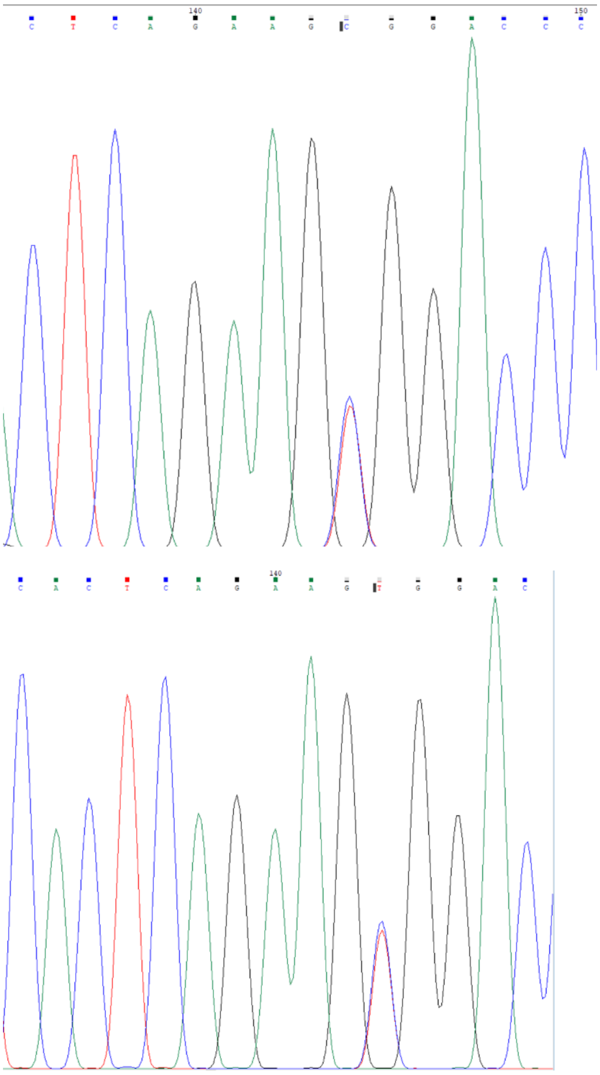

UBOX5 p.Y493S

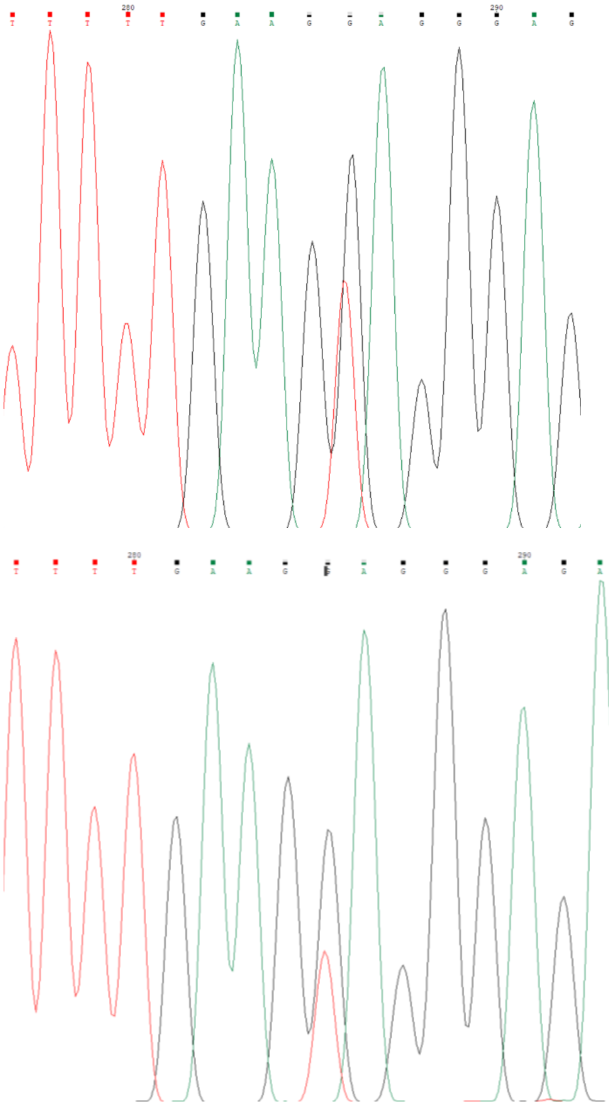

UBOX5 p.E482K

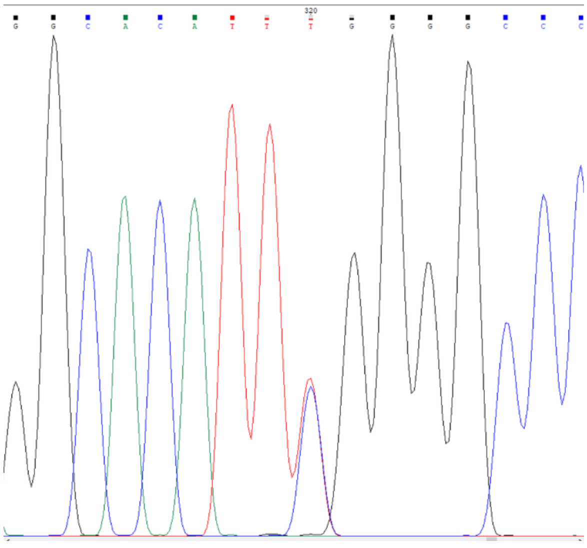

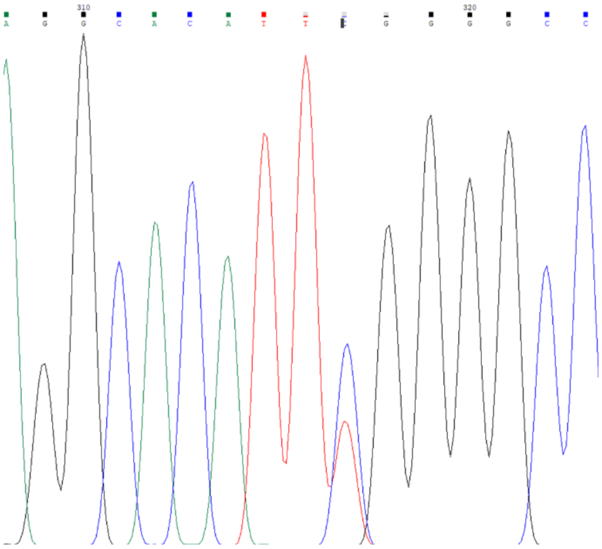

UBOX5 p.A434T

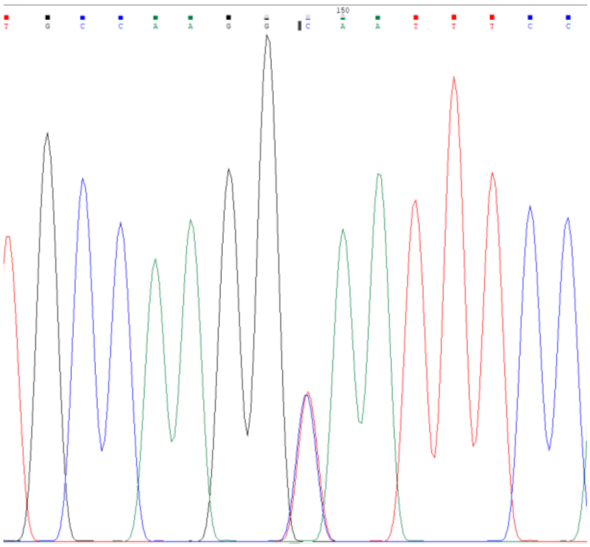

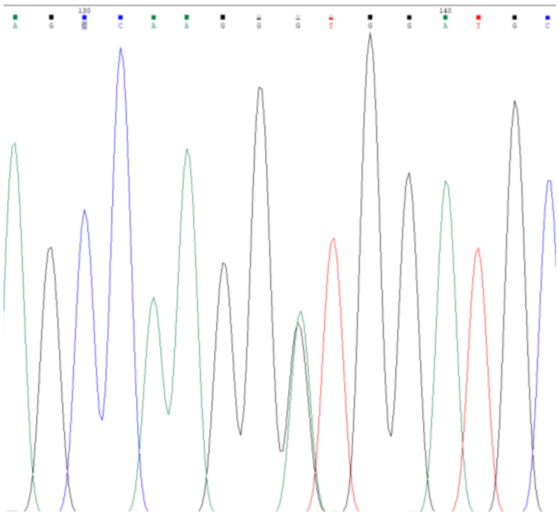

Supplementary Figure 12: Exome-wide variant count distribution for the discovery exome sequencing series for each constituent dataset, stratified by cases and controls.

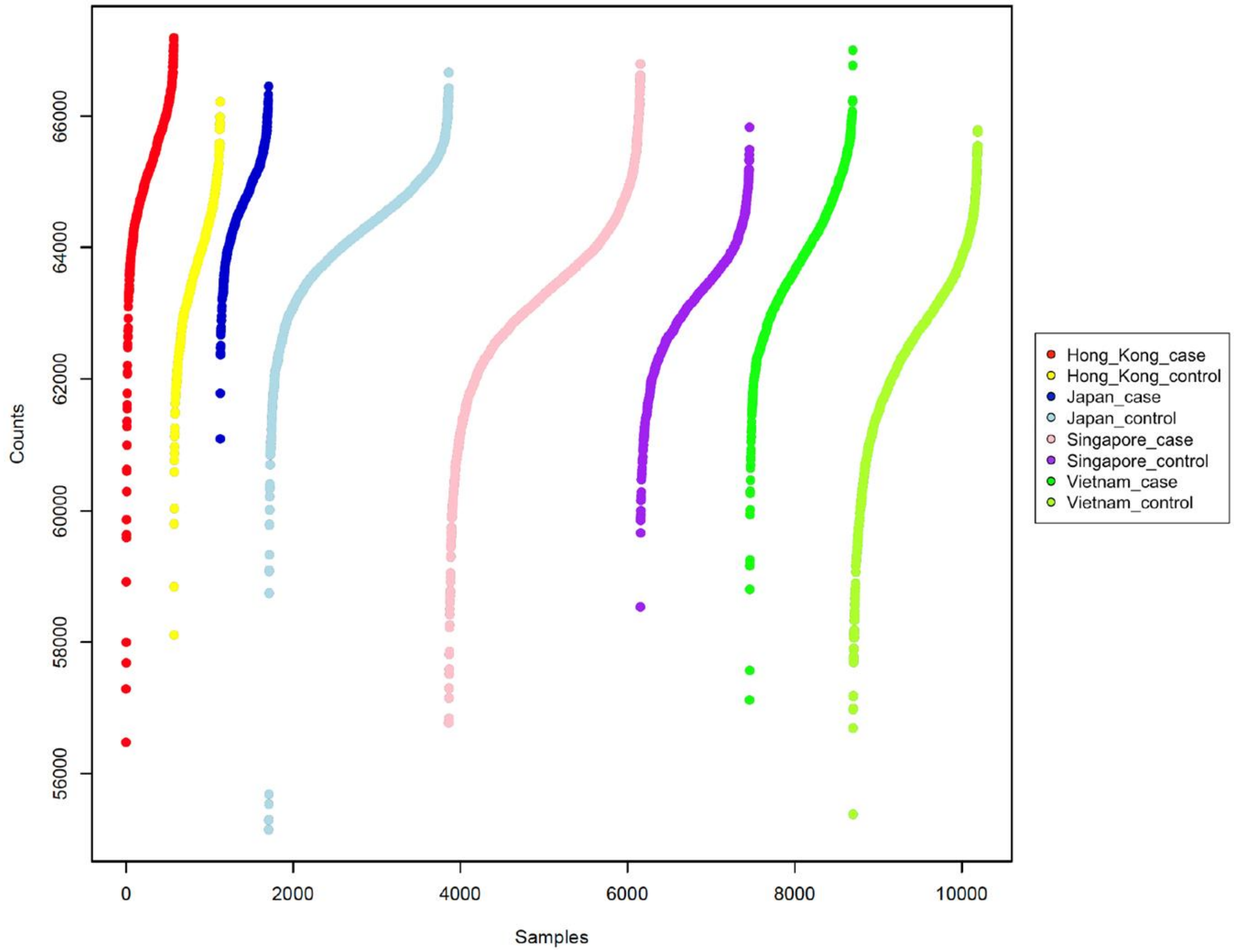

Supplementary Figure 13: FLAG-tagged UBOX5 (3 lanes on the right) or FLAG-tagged UBOX5- UBD construct (4 lanes on the left) and HA-tagged ubiquitin was co-transfected into HEK293 cells. After the first immunoprecipitation of lysates by anti-FLAG antibody, 20% of eluates was kept for analysis. Eluate from the UBOX5-UBD was further immunoprecipitated with anti-HA antibody to enrich for ubiquitinated proteins. To serve as antibody specificity control (negative controls), lysates were mock immunoprecipitated with mouse immunoglobulin (IgG). Immunoblotting of HA or FLAG was performed on inputs and eluates as indicated. Bands corresponding to ubiquitinated UBOX5 and ubiquitinated UBOX5-UBD chimeric protein is indicated by vertical lines, while the unmodified proteins are indicated by arrows on the right. Positions of the molecular weight markers are indicated by arrows on the left. IB:HA showed smeared bands because a multitude of ubiquitinated proteins (and not just BIP) were bound to it.

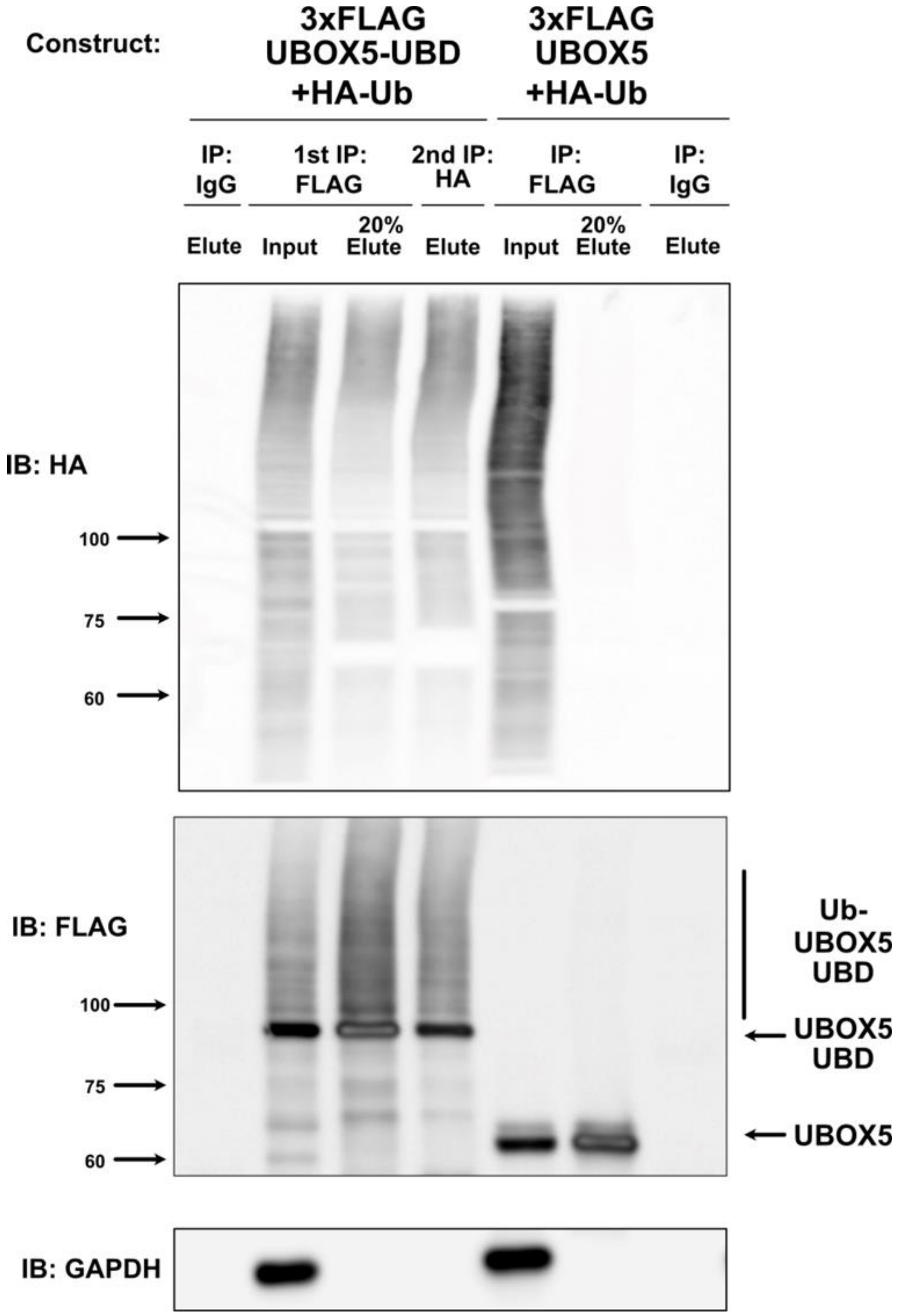

Supplementary Figure 14: Quantile-quantile plot of the discovery exome sequencing series for gene-based burden of rare synonymous alleles.

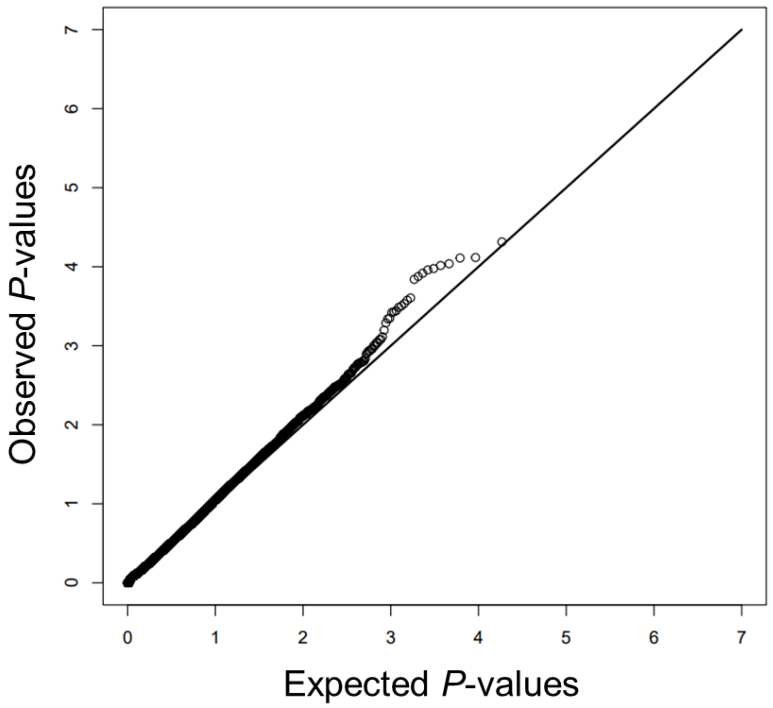

Supplementary Figure 15: Risk of primary angle-closure glaucoma with *UBOX5* synonymous variants.

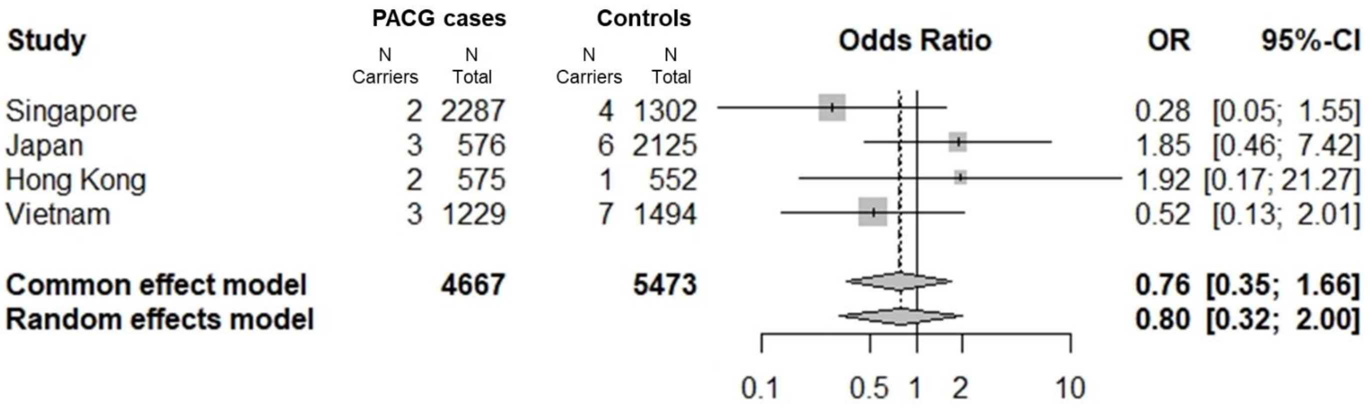

Supplementary Figure 8 uncropped immunoblots

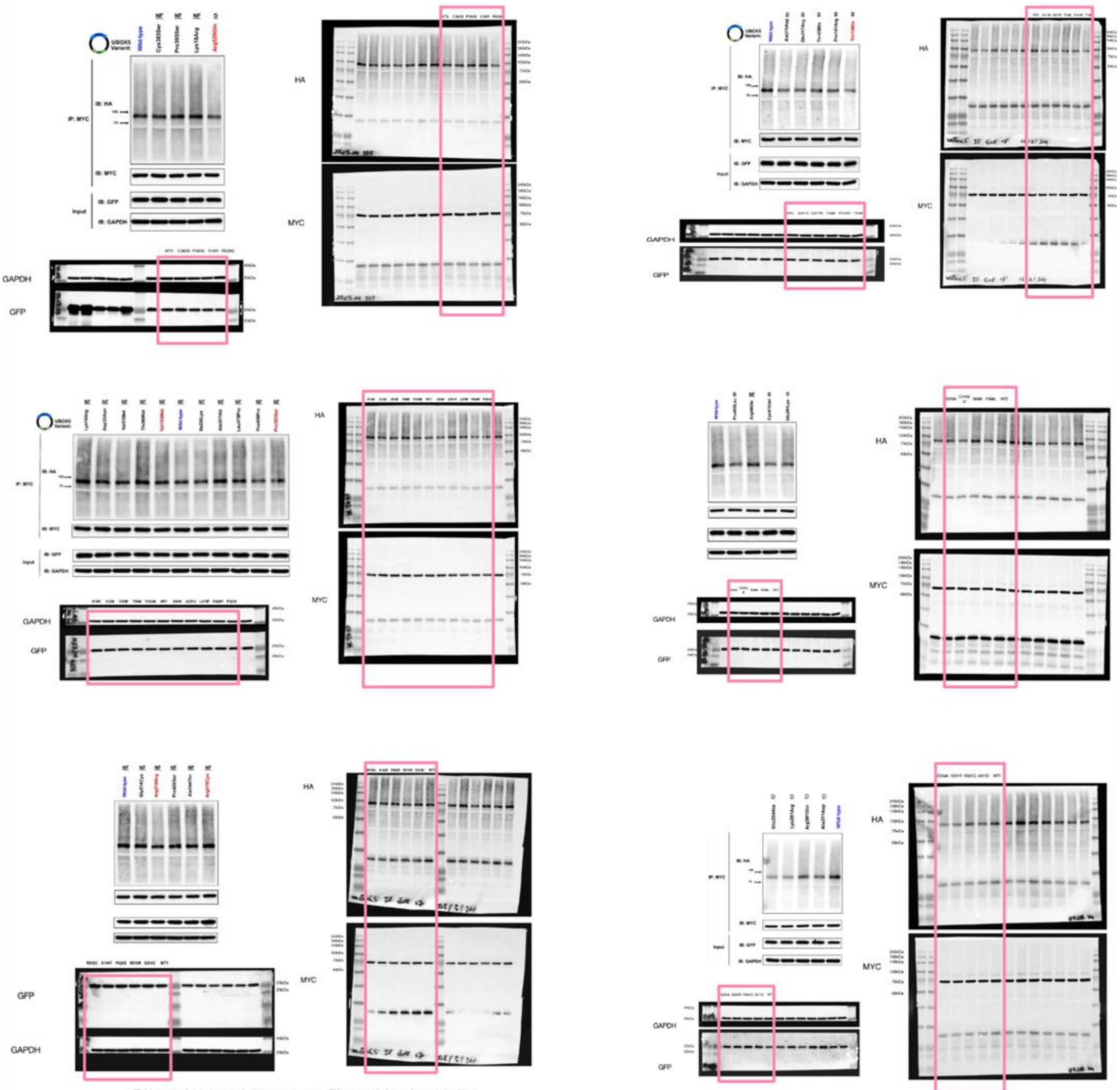

Blots shown above were flipped horizontally

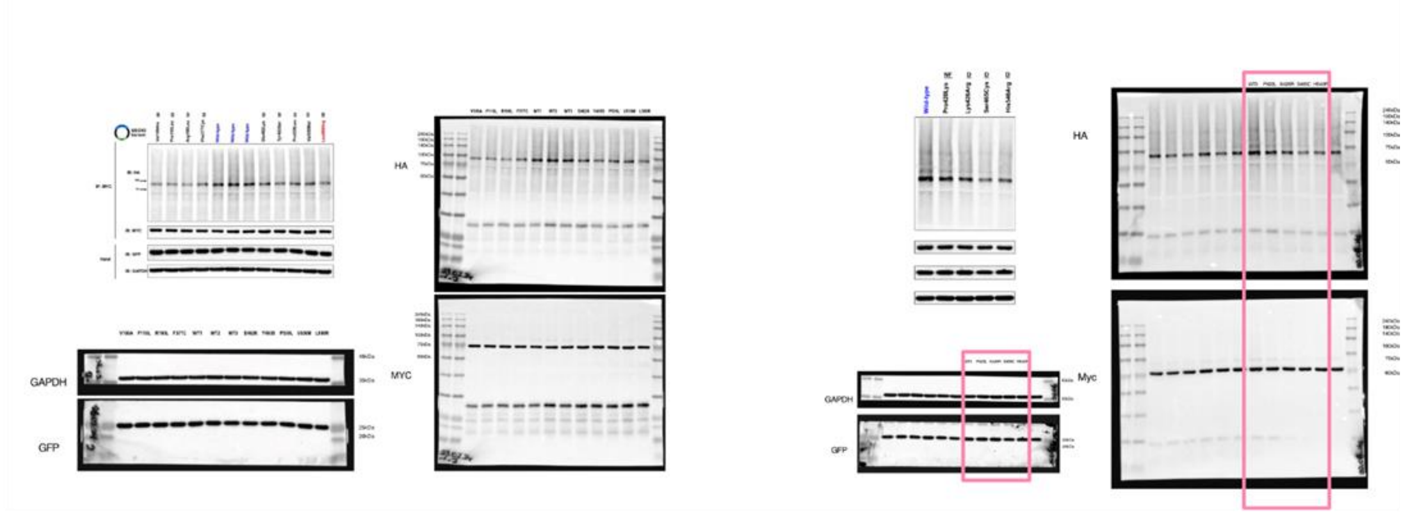

Supplementary References

1 Genetics of Exfoliation Syndrome, P. *et al.* Association of Rare CYP39A1 Variants With Exfoliation Syndrome Involving the Anterior Chamber of the Eye. *Jama* **325**, 753-764, doi:10.1001/jama.2021.0507 (2021).

2 Cirulli, E. T. *et al.* Exome sequencing in amyotrophic lateral sclerosis identifies risk genes and pathways. *Science* **347**, 1436-1441, doi:10.1126/science.aaa3650 (2015).

3 Do, R. *et al.* Exome sequencing identifies rare LDLR and APOA5 alleles conferring risk for myocardial infarction. *Nature* **518**, 102-106, doi:10.1038/nature13917 (2015).

4 Kircher, M. *et al.* A general framework for estimating the relative pathogenicity of human genetic variants. *Nature genetics* **46**, 310-315, doi:10.1038/ng.2892 (2014).

5 Vihinen, M. When a Synonymous Variant Is Nonsynonymous. *Genes (Basel)* **13**, doi:10.3390/genes13081485 (2022).

6 Khor, C. C. *et al.* Genome-wide association study identifies five new susceptibility loci for primary angle closure glaucoma. *Nature genetics* **48**, 556-562, doi:10.1038/ng.3540 (2016).

7 Wu, M. C. *et al.* Rare-variant association testing for sequencing data with the sequence kernel association test. *American journal of human genetics* **89**, 82-93, doi:10.1016/j.ajhg.2011.05.029 (2011).
