# Supplementary material for: Functionally deficient UBOX5 variants and primary angle-closure glaucoma
Source: Nat Commun. 2025 Aug 15;16:7620. doi: 10.1038/s41467-025-62775-x (PMC12356834; doi:10.1038/s41467-025-62775-x)
Supplement: Supplementary file 2 — Description of Additional Supplementary Files [file 41467_2025_62775_MOESM2_ESM.pdf]

Description of Additional Supplementary Files

Supplementary Data 1: Summary statistics for the discovery whole exome sequencing study from Hong Kong, Japan, Singapore, and Vietnam

QV-cases: Number of qualifying rare variants* in persons with primary angle-closure glaucoma

QV-controls: Number of qualifying rare variants in unaffected individuals

NonQV-cases: Number of persons with primary angle-closure glaucoma who were not carrying qualifying rare variants.

NonQV-controls: Number of unaffected individuals who were not not carrying qualifying rare variants.

Odds Ratio: Odds of primary angle-closure glaucoma for carriers of qualifying rare variants compared to non-carriers; calculated for each gene from the gene-based burden test.

L95: Lower boundary of the 95% confidence interval of the Odds Ratio

U95: Upper boundary of the 95% confidence interval of the Odds Ratio

$\boldsymbol{P}$-$_{\text{CMH}}$: *P*-value obtained from stratified meta-analysis of Hong Kong, Japan, Singapore, and Vietnam case-control studies using the Cochran Mantel-Haenszel method

$\boldsymbol{P}$-$_{\text{PCA+VC}}$: *P*-value obtained from meta-analysis of Hong Kong, Japan, Singapore, and Vietnam case-control studies after adjusting for ancestry principal components and exome-wide variant count (total exome variant count).

Genes surpassing exome-wide significance ($P < 2.5 \times 10^{-6}$) are highlighted in red.

*Qualifying rare variants are defined as mis-sense variants with allele frequency less than 1% and having a CADD score >10. Variants with such a CADD score are predicted to be within the top 10% most deleterious substitutions that can occur in the human genome.
