# Supplementary material for: Functionally deficient UBOX5 variants and primary angle-closure glaucoma
Source: Nat Commun. 2025 Aug 15;16:7620. doi: 10.1038/s41467-025-62775-x (PMC12356834; doi:10.1038/s41467-025-62775-x)
Supplement: Supplementary file 4 — Reporting Summary [file 41467_2025_62775_MOESM4_ESM.pdf]

nature portfolio

Corresponding author(s): Chiea Chuen Khor and Zhenxun Wang

Last updated by author(s): Jul 3, 2025

Reporting Summary

Nature Portfolio wishes to improve the reproducibility of the work that we publish. This form provides structure for consistency and transparency in reporting. For further information on Nature Portfolio policies, see our Editorial Policies and the Editorial Policy Checklist.

Statistics

For all statistical analyses, confirm that the following items are present in the figure legend, table legend, main text, or Methods section.

n/a	Confirmed	
☐	☒	The exact sample size (n) for each experimental group/condition, given as a discrete number and unit of measurement
☒	☐	A statement on whether measurements were taken from distinct samples or whether the same sample was measured repeatedly
☐	☒	The statistical test(s) used AND whether they are one- or two-sided<br>*Only common tests should be described solely by name; describe more complex techniques in the Methods section.*
☐	☒	A description of all covariates tested
☐	☒	A description of any assumptions or corrections, such as tests of normality and adjustment for multiple comparisons
☐	☒	A full description of the statistical parameters including central tendency (e.g. means) or other basic estimates (e.g. regression coefficient) AND variation (e.g. standard deviation) or associated estimates of uncertainty (e.g. confidence intervals)
☐	☒	For null hypothesis testing, the test statistic (e.g. F, t, r) with confidence intervals, effect sizes, degrees of freedom and P value noted<br>*Give P values as exact values whenever suitable.*
☒	☐	For Bayesian analysis, information on the choice of priors and Markov chain Monte Carlo settings
☒	☐	For hierarchical and complex designs, identification of the appropriate level for tests and full reporting of outcomes
☐	☒	Estimates of effect sizes (e.g. Cohen's d, Pearson's r), indicating how they were calculated

Our web collection on statistics for biologists contains articles on many of the points above.

Software and code

Policy information about availability of computer code

Data collection	Data collection was from Illumina high-throughput, short read sequencers, as well as from clinical observations from glaucoma specialists. In this light, no computer code was used for data collection.
Data analysis	Burrow-Wheeler Aligner software (version 0.7.17-r1188) for mapping sequence reads. Genome Analysis Tool Kit (version 4.1.3.0) for variant calling. VCFTOOLS (version 0.1.16), BCFTOOLS (version 1.18), and PLINK (v2.00) for quality control of genetic data. The ENSEMBL VEP (variant effect predictor)(release 110, grch37) was used to annotate variants.

For manuscripts utilizing custom algorithms or software that are central to the research but not yet described in published literature, software must be made available to editors and reviewers. We strongly encourage code deposition in a community repository (e.g. GitHub). See the Nature Portfolio guidelines for submitting code & software for further information.

Data

Policy information about availability of data

All manuscripts must include a data availability statement. This statement should provide the following information, where applicable:

- Accession codes, unique identifiers, or web links for publicly available datasets
- A description of any restrictions on data availability
- For clinical datasets or third party data, please ensure that the statement adheres to our policy

The exome-wide summary statistics for the discovery whole exome sequencing analysis are appended in the Supplementary Dataset. The discovery whole exome sequencing dataset in PLINK format have been deposited in the NGDC OMIX archive under accession ID OMIX007093. The dataset is available under restricted

access due to the sensitive nature of individual level genotypes from whole-exome sequencing data, access can be obtained by requesting via the NGDC OMIX archive website (https://ngdc.cncb.ac.cn/omix/releaseList).

Research involving human participants, their data, or biological material

Policy information about studies with human participants or human data. See also policy information about sex, gender (identity/presentation), and sexual orientation and race, ethnicity and racism.

Reporting on sex and gender	Primary analysis evaluating the association between genetic variants and presence of primary angle-closure glaucoma (PACG) was performed on all participants without consideration of sex. Experiment-wide statistical significance was observed at a gene named UBOX5. For UBOX5, we performed post-hoc, secondary analysis stratifying by sex to evaluate whether the association between carriers of rare, protein-altering variants were associated with stronger risk of PACG in either sex. We also stratified the analysis by sex to evaluate the association between UBOX5 rare variants and risk of acute primary angle-closure. All numbers of participants with PACG and unaffected controls involved in the primary analysis, together with the numbers of male and female participants involved in the secondary analysis are shown in Figure 1.
Reporting on race, ethnicity, or other socially relevant groupings	We did not specifically report on race, ethnicity, or other socially relevant groupings in this study, other than noting in the introduction section of the manuscript that Primary angle-closure glaucoma (PACG) is a major form of glaucoma that disproportionately affects persons of Asian ancestry.
Population characteristics	see above
Recruitment	All participants were recruited after written informed consent, adhering to the Declaration of Helsinki. The studies were approved by all relevant local and hospital Institutional Review Boards. The inclusion criteria for patients with primary angle-closure glaucoma (PACG) were:<br>a) Patients with previous Acute primary angle closure and/or b) Patients with chronic PACG.<br>Patients younger than 50 years were excluded, as were patients with secondary forms of angle closure glaucoma such as neovascular glaucoma. As far as possible, unaffected control individuals were participants ≥50 years old and an eye examination to confirm the absence of glaucoma.
Ethics oversight	The hospital Institutional Review Boards for each participating hospital (shown in the Supplementary Information) approved the studies.

Note that full information on the approval of the study protocol must also be provided in the manuscript.

Field-specific reporting

Please select the one below that is the best fit for your research. If you are not sure, read the appropriate sections before making your selection.

☒ Life sciences ☐ Behavioural & social sciences ☐ Ecological, evolutionary & environmental sciences

For a reference copy of the document with all sections, see nature.com/documents/nr-reporting-summary-flat.pdf

Life sciences study design

All studies must disclose on these points even when the disclosure is negative.

Sample size	Our study design had an estimated statistical power of 88% to surpass exome-wide significance (preset as P<2.5×10-6 to account for gene-based burden tests on the ≈20,000 genes found in the human genome) in the discovery exome sequencing analysis. This power calculation (shown in Supplementary Table 5) applies to all genes with cumulative rare variant burden of at least 1 percent, with a differential variant burden between cases and controls associated with an odds ratio (OR) of at least 2.0. Considering other scenarios, statistical power remains adequate for discovering genes at the exome-wide significance threshold for which either the differential variant burden was associated with a large effect size (e.g. ORs ≥2.5 could be detected for genes with cumulative rare variant burden as low as 0.5%, at 86.8% power), or genes for which large numbers of qualifying rare variant carriers are observed (e.g. genes with a minimum cumulative rare variant burden of 2% could be detected with an OR ≥1.7 at >90% power).
Data exclusions	Samples with genotyping completion rate of less than 95% were excluded, as the inclusion of such samples may result in biased / artifactual findings that may not be reproducible. Likewise, genetic variants with completion rate of less than 95% were also excluded from further analysis.
Replication	To replicate statistically significant findings (defined as exome-wide significance, P<2.5x10-6) from the discovery exome sequencing study, ten additional case-control panels independent from the discovery study were evaluated. The first nine panels comprised 760 persons with primary angle-closure glaucoma (PACG) and 3,844 unaffected individuals ascertained from hospital-based studies, and the tenth was from the community-based UK Biobank. In the UK Biobank, 469,639 participants underwent exome sequencing, of which 1,759 had PACG.
Randomization	This is not relevant to our study, as our study is not a randomized clinical trial. Instead, our study is a case-control genetic association study whereby affected individuals (persons with primary angle closure glaucoma) and unaffected individuals (unaffected control persons) with known disease status were analyzed.
Blinding	We ensured that DNA from persons with PACG and DNA from controls were prepared using the same exome capture kit as far as possible. Sequencing reads were mapped in a manner blinded to case-control status of the participants, and variant calling was also performed blinded to case-control status.

Reporting for specific materials, systems and methods

We require information from authors about some types of materials, experimental systems and methods used in many studies. Here, indicate whether each material, system or method listed is relevant to your study. If you are not sure if a list item applies to your research, read the appropriate section before selecting a response.

Materials & experimental systems

n/a	Involved in the study
☐	☒ Antibodies
☐	☒ Eukaryotic cell lines
☒	☐ Palaeontology and archaeology
☒	☐ Animals and other organisms
☒	☐ Clinical data
☒	☐ Dual use research of concern
☒	☐ Plants

Methods

n/a	Involved in the study
☒	☐ ChIP-seq
☒	☐ Flow cytometry
☒	☐ MRI-based neuroimaging

Antibodies

Antibodies used

Anti-c-Myc (9E10) (Santa Cruz, #sc-40, lot #C2224) was used for the immunoprecipitation (IP) pull-down of the UBOX5 and its variants.
Anti-FLAG (M2) (Sigma Aldrich, #F1804, lot #SLCM4081) and anti-HA (Roche, #11666606001) were used for the tandem IP pull-down.
Anti-HA (Proteintech, #51064-2-AP, lot #00061770), anti-c-Myc (A-14) (Santa Cruz, #sc-789, lot #C1314), anti-GRP78/BIP (Proteintech, #11587-1-AP, lot #00114059), anti-UBOX5 (Novus Biologicals, #NBP1-81469, lot #R30897), anti-GAPDH (0411) (Santa Cruz, #47724, lot #H2521), and anti-GFP (Abcam, #ab13970, lot #1018753-2) were used for immunoblotting (IB).
Custom anti-mouse UBOX5 (lot #37002-37004) was used for immunohistochemistry (IHC), immunofluorescence (IF), and IB of mice sections and NIH-3T3 cell line. Custom anti-mouse ubox5 (Genemed, lot #37002-37004)

Validation

Anti-HA (#11666606001), anti-HA (#51064-2-AP), and anti-UBOX5 (#NBP1-81469) have been validated in our immunoblots on HEK 293T cell line with gene overexpression and negative controls in Figures 3B-C in the manuscript main text.
Anti-c-Myc (9E10) (#sc-40), anti-c-Myc (A-14) (#sc-789), anti-FLAG (M2) (#F1804), anti-GAPDH (0411) (#47724), and anti-GFP (#ab13970) have been validated by the respective manufacturers (Santa Cruz, Sigma Aldrich, Abcam) with multiple citations.
Anti-GRP78/BIP (#11587-1-AP) has been KD/KO validated by manufacturer (Proteintech) with multiple citations.
Custom anti-mouse UBOX5 (lot #37002-37004) has been validated through IF of mice sections with UBOX5 knockout mice (do please refer to Supplementary Figure 5).

Eukaryotic cell lines

Policy information about cell lines and Sex and Gender in Research

Cell line source(s)	All cell lines, HEK 293T (CRL-3216) and NIH/3T3 (CRL-1658) were purchased from ATCC.
Authentication	None of the cell lines were authenticated, early passage cell lines were used.
Mycoplasma contamination	All cell lines tested negative for mycoplasma contamination.
Commonly misidentified lines (See ICLAC register)	This is not relevant to our study.

Plants

Seed stocks	This study did not involve plants.
Novel plant genotypes	This study did not involve plants.
Authentication	This study did not involve plants.
