# Supplementary figures and images for: Functionally deficient UBOX5 variants and primary angle-closure glaucoma
Source: Nat Commun. 2025 Aug 15;16:7620. doi: 10.1038/s41467-025-62775-x (PMC12356834; doi:10.1038/s41467-025-62775-x)

**Figure 2A**

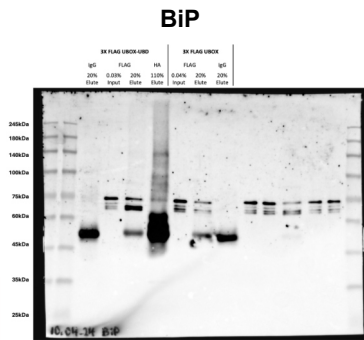

**Figure 2B**

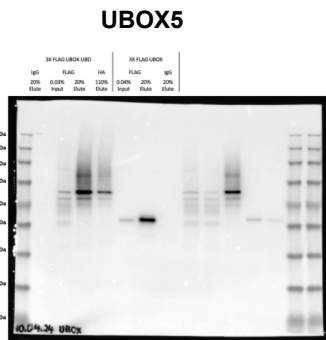

HA

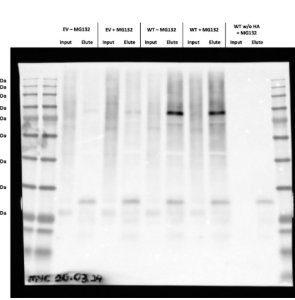

**Myc**

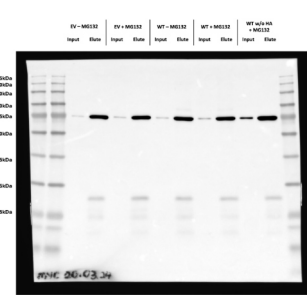

**Figure 2C**

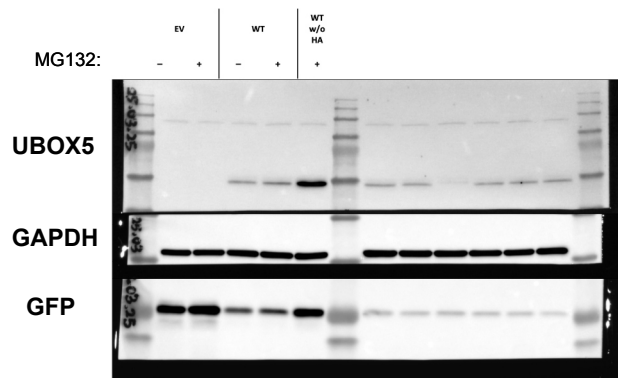

**Figure 3A**

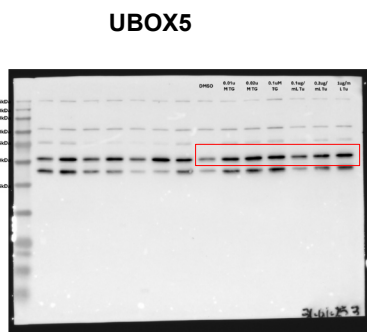

**beta-actin**

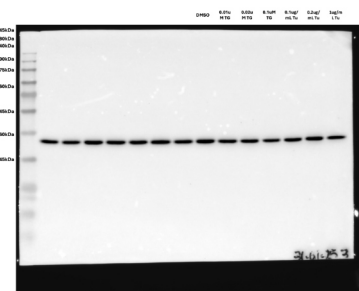

**Figure 3B**

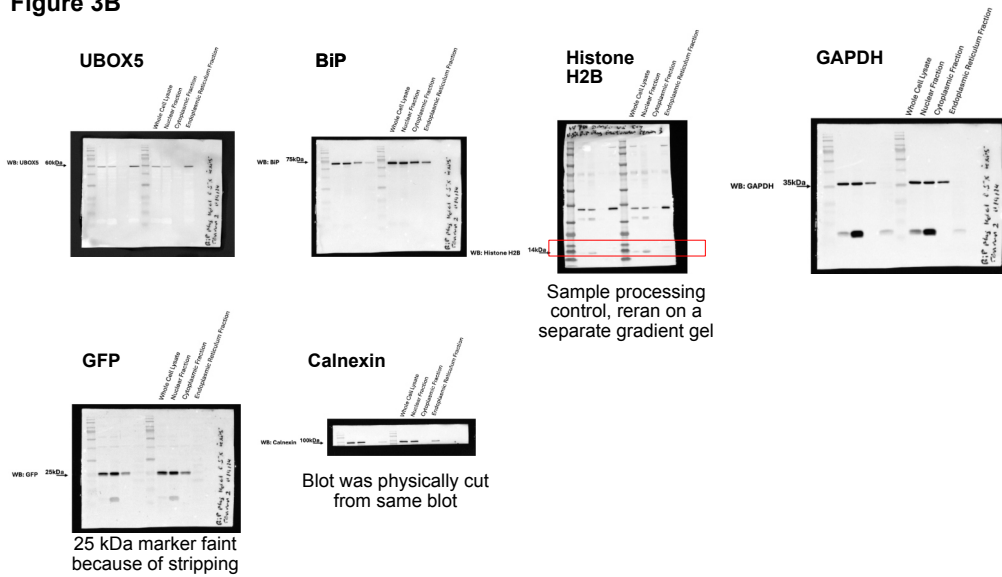

**Figure 3C**

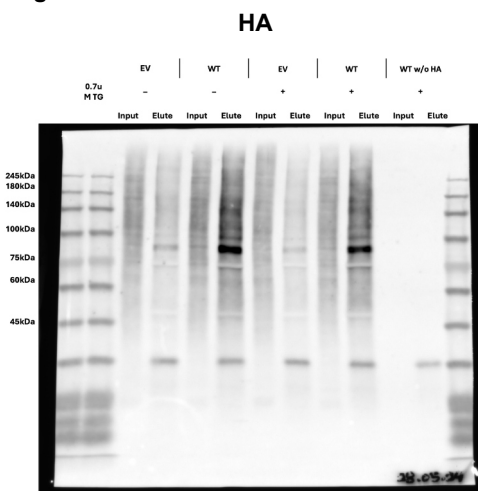

## Myc

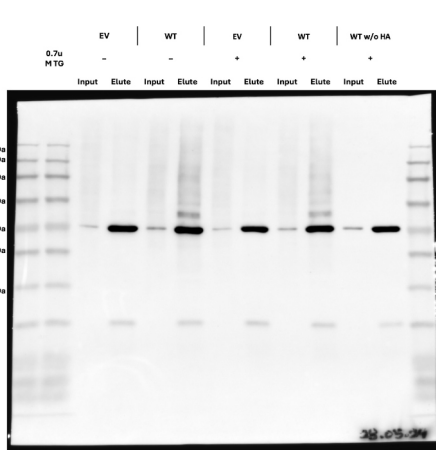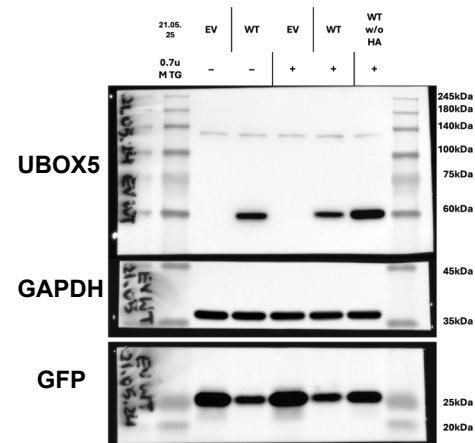

Figure 5A

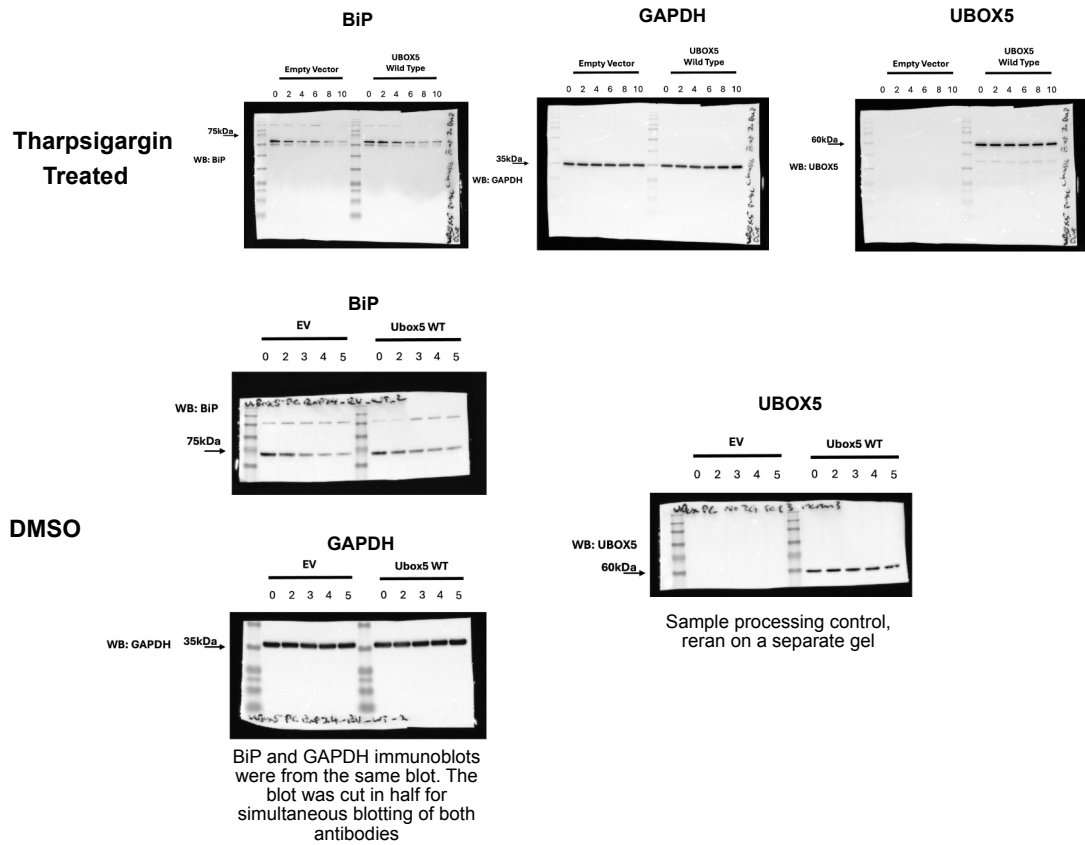

Figure 5B

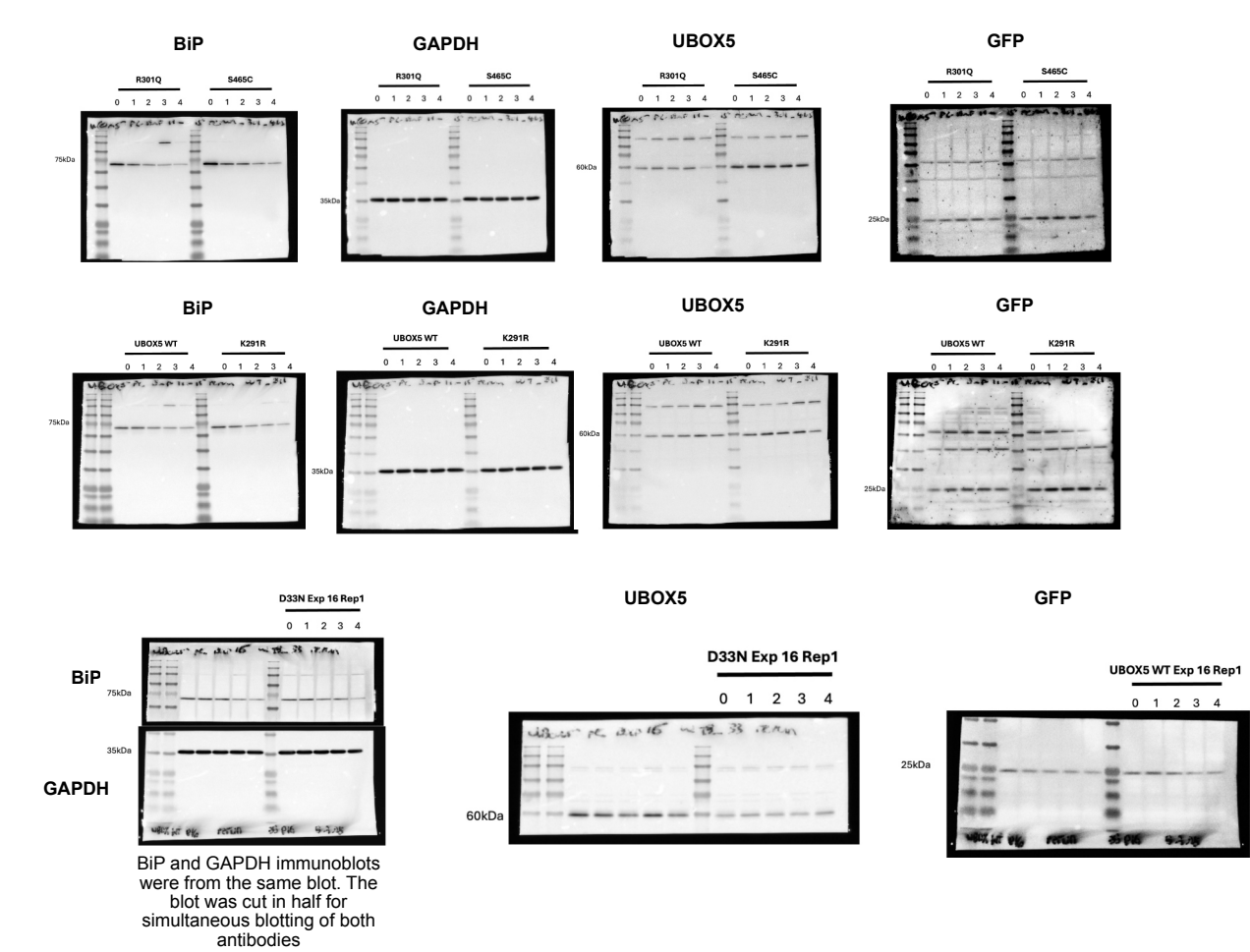

Supplement: Supplementary file 6 — Source Data 1 [file 41467_2025_62775_MOESM6_ESM.zip › Source Data File for NCOMMS-24-46851B.pdf]

**Highlighted Samples are used for quantification**

**Same color – same set of quantification**



250106

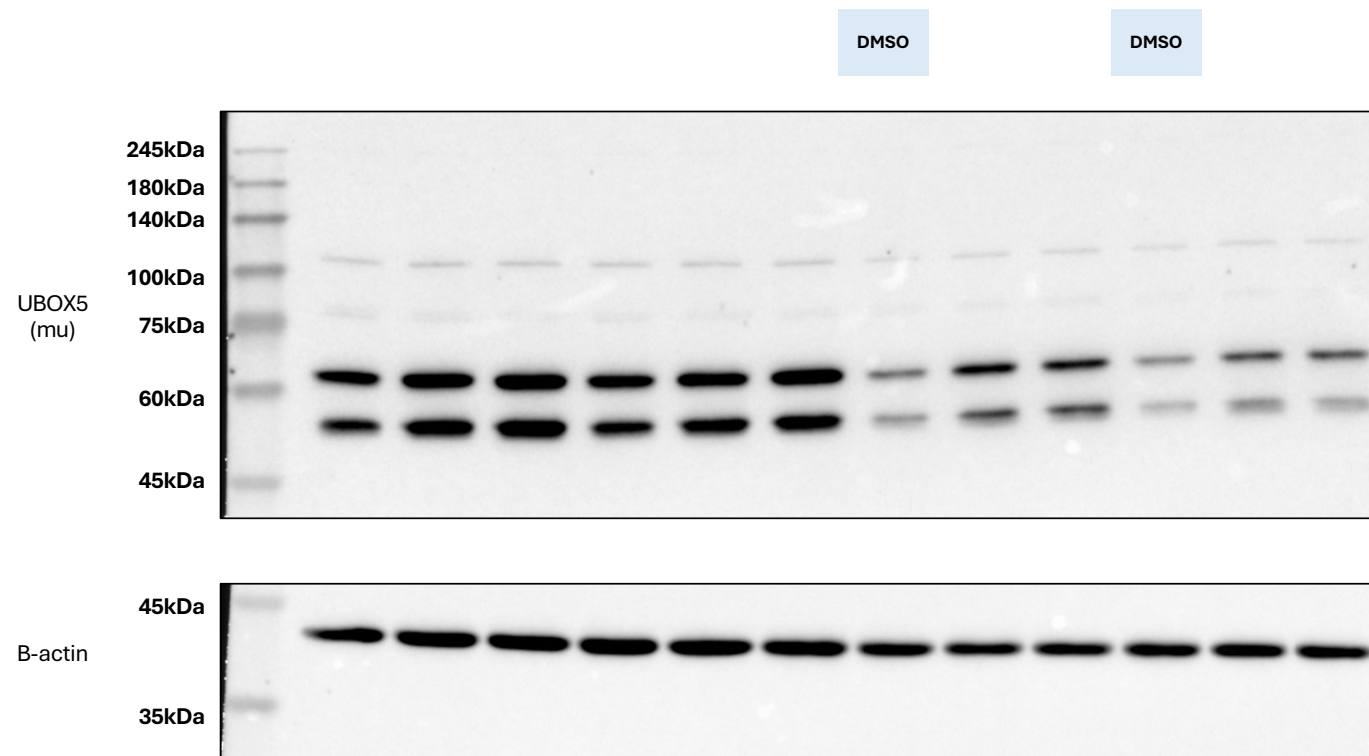

250110

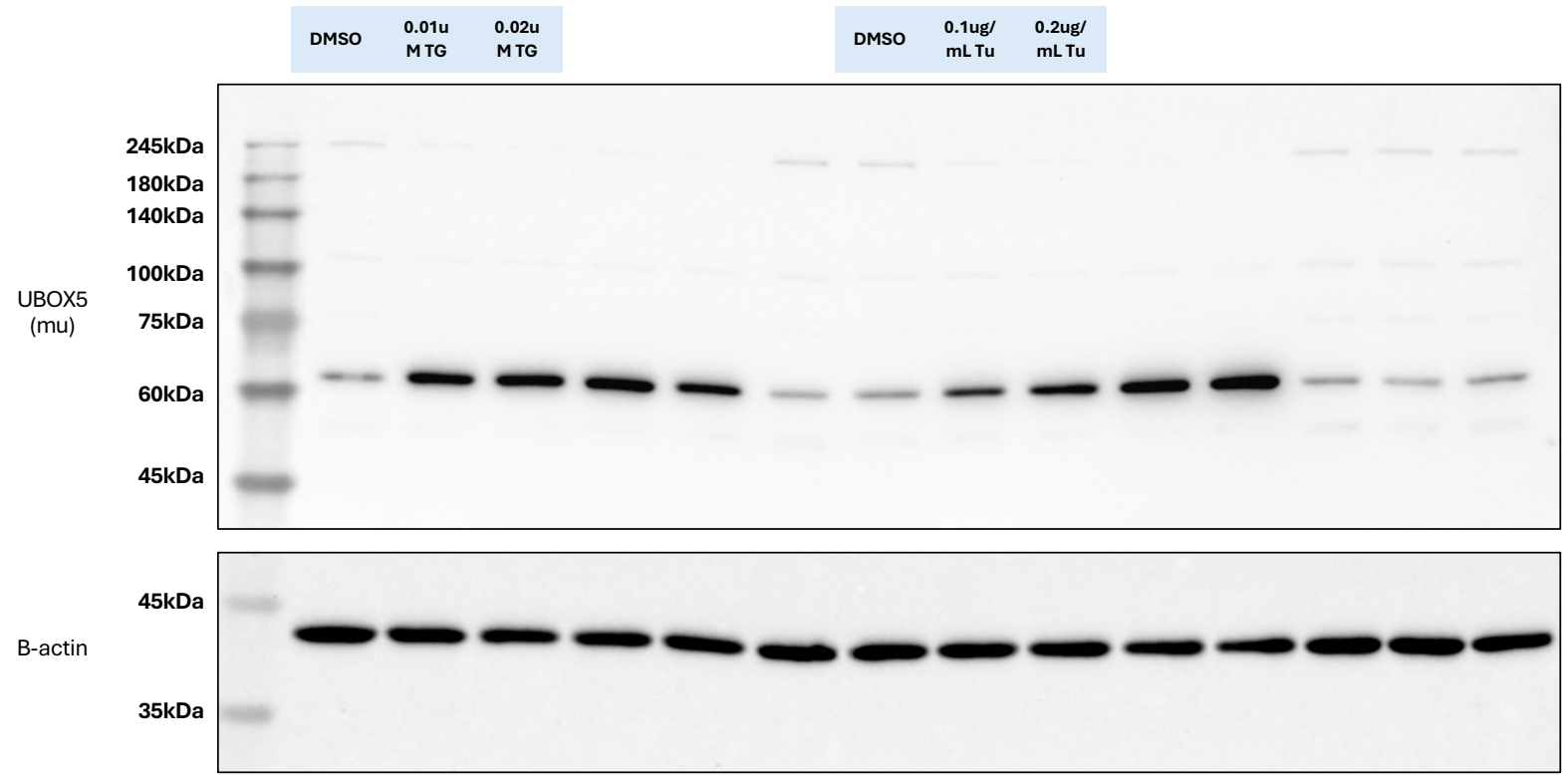

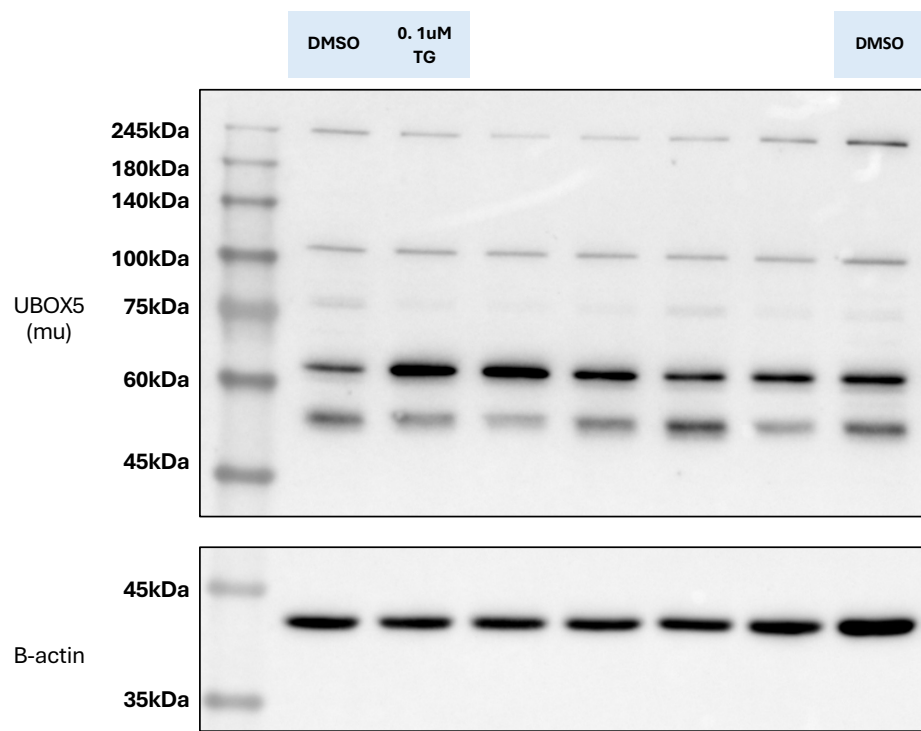

250131

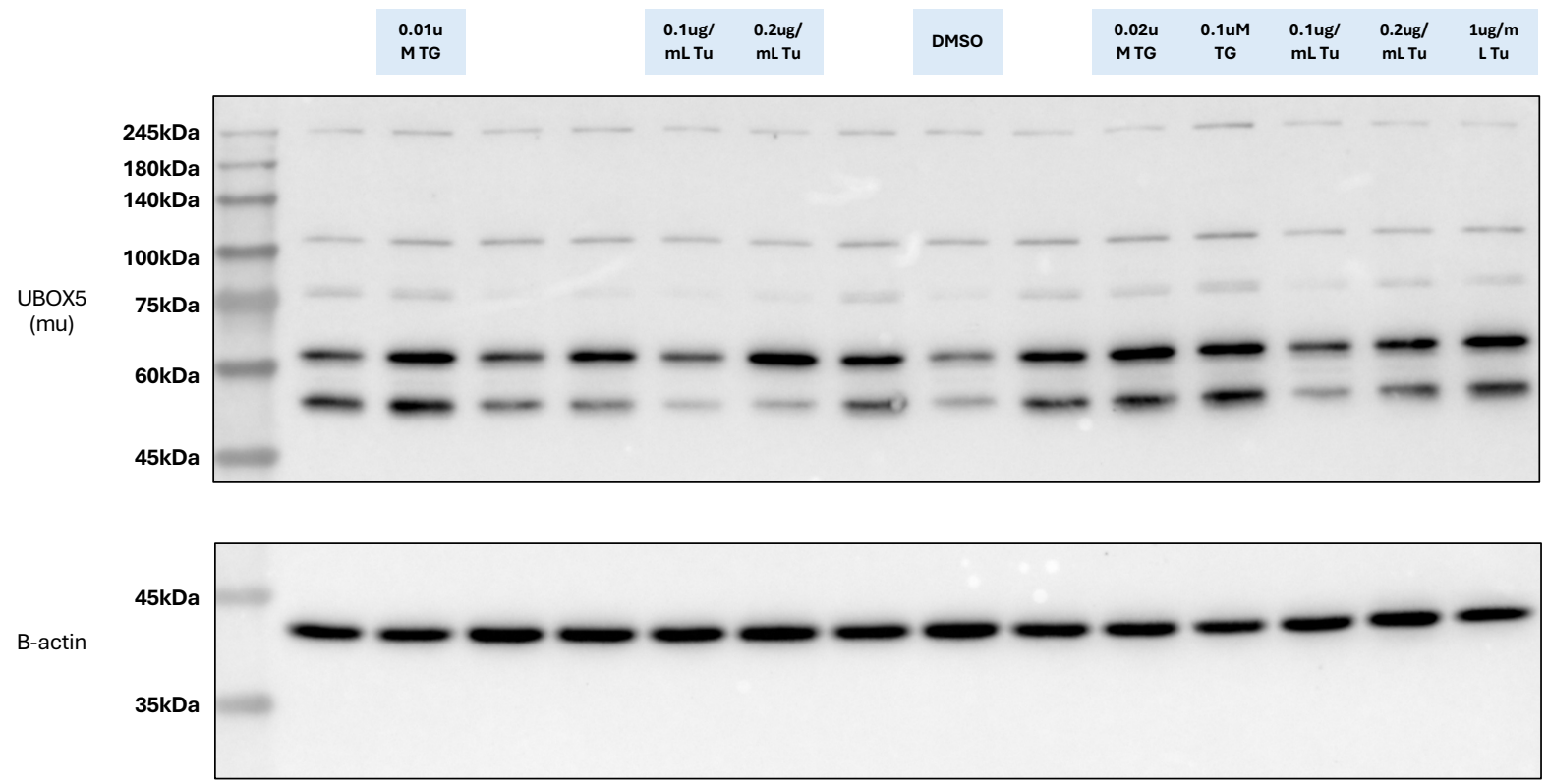

250207

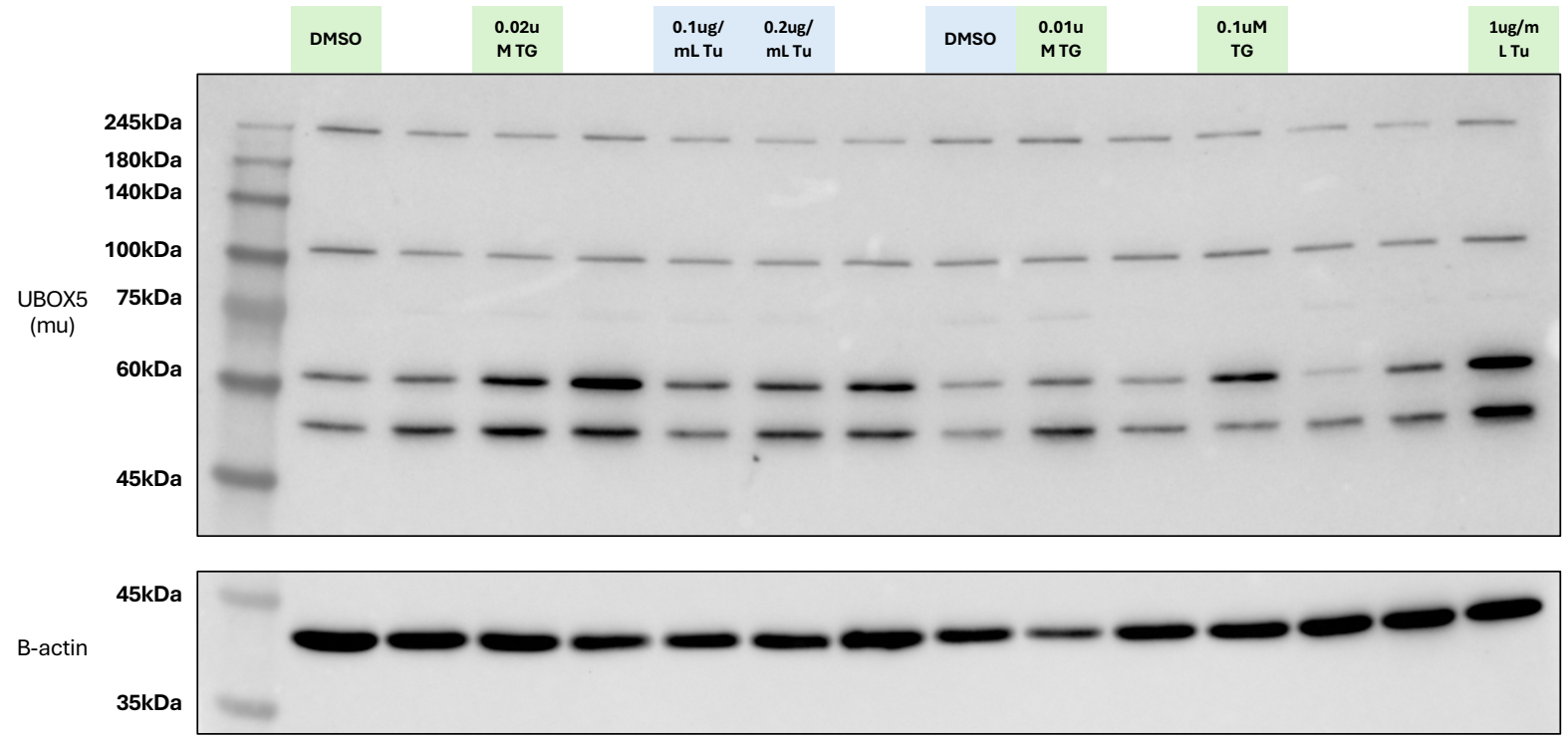

Supplement: Supplementary file 7 — Source Data 2 [file 41467_2025_62775_MOESM7_ESM.zip › Figure 3A quantitation files Final/Gel Images for Figure 3A WB Quantitation.pdf]
